# Antibacterial and antioxidant activities of extracts and isolated compounds from the roots extract of *Cucumis prophetarum* and in silico study on DNA gyrase and human peroxiredoxin 5

**DOI:** 10.1186/s13065-021-00758-x

**Published:** 2021-05-06

**Authors:** Wario Galma, Milkyas Endale, Emebet Getaneh, Rajalakshmanan Eswaramoorthy, Temesgen Assefa, Yadessa Melaku

**Affiliations:** 1 Department of Applied Chemistry, School of Applied Natural Science, Adama Science and Technology University, P.O.Box 1888, Adama, Ethiopia; 2 Department of Biotechnology, College of Natural and Computational Science, Debre Birhan University, P.O. Box 445, Debre Birhan, Ethiopia

**Keywords:** *C. prophetarum*, Antibacterial, Antioxidant, Docking studies

## Abstract

**Background:**

*Cucumis prophetarum* is traditionally used to treat liver and lung disorders, heart failure, diarrhea, gonorrhea, skin infections, intestinal problems and cancer. In the present work, the isolation of two novel compounds along with their antibacterial and antioxidant activities is reported for the first time.

**Methods:**

Silica gel column chromatography was applied to separate constituents of the roots of *C. prophetarum.* The structures of isolated compounds were established using ^1^H NMR, ^13^C NMR, DEPT-135, COSY, HSQC and HMBC. Agar well diffusion, DPPH assay and ferric thiocyante methods were used for antibacterial, radical scavenging and anti-lipid peroxidation activities, respectively. AutoDock Vina open source program was used for molecular docking analysis.

**Results:**

Evaluation of the in vitro antibacterial activity of the constituents against *S. aureus, B. subtilis, E. coli* and *S. thyphimurium* revealed that the hexane extract were active against *E. coli* with IZ of 15.0 ± 1.41 mm, whereas an IZ of 14.6 ± 1.70 mm for MeOH extract was observed against *S. aureus*. Compound **1** displayed IZ of 13.6 ± 0.94 mm against *E. coli* and curcurbiatin **2** showed activity against *B. subtilis* with IZ of 13.3 ± 0.54 mm. The molecular docking analysis showed that cucurbitacins **2** and **3** have binding energy of -6.7 and -6.9 kcal/mol, respectively. The methanol and the hexane extracts of the roots of *C. prophetarum* inhibited DPPH radical by 70.4 and 63.3% at 100 µg/mL, respectively. On the other hand, the methanol extract inhibited lipid peroxidation by 53.0%.

**Conclusion:**

The present study identified five compounds from the root extracts of *C. prophetarum*, of which two are novel cucurbitacins (**1**, **2**). The in vitro antibacterial activity of the hexane and methanol extracts was better than the activity displayed by the isolated compounds. This is probably due to the synergistic effects of the constituents present in the root extract. The in silico molecular docking study results showed that, compounds **2** and **3** have minimum binding energy and have good affinity toward the active pocket, thus, they may be considered as good inhibitor of DNA gyrase B. Furthermore, the “drug-likeness” and ADMET prediction of compounds **2–5** nearly showed compliance with the Lipinski rule, with good absorption, distribution, metabolism, and excretion generally. The radical scavenging and anti-lipid peroxidation activities of the extracts were better than the isolated compounds. This is attributed to the presence of phenolics and flavonoids as minor constituents in the extracts of these species. Therefore, the in vitro antibacterial activity and molecular docking analysis suggest the potential use of the isolated compounds as medicine which corroborates the traditional use of the roots of *C. prophetarum*.

**Supplementary Information:**

The online version contains supplementary material available at 10.1186/s13065-021-00758-x.

## Introduction

Cucurbitacins are chemically characterized as a group of highly oxygenated tetracyclic triterpenes widely found in many medicinal plants [1]. They are produced by plants to defend themselves from external biological predators and parasites [2]. Various reports demonstrated that cucurbitacin and its analogues have wide arrays of pharmacological properties including anticancer, antitumor, antipyretic, analgesic, antiinflammatory, and hepatoprotective effects [3–5]. Based on their structural characteristics, cucurbitacins are categorized into 12 classes and designated alphabetically from A to T with over 200 derivatives were reported so far [6]. Cucurbitacins are usually crystalline in nature, purgative, hydrophobic and soluble in organic solvents [7]. They occur in various families and genera of plants, but commonly found in *Cucumis* which have been used in conventional medicine for decades [8]. Some plants of the genus *Cucumis* are used as traditional remedies as antiinflammatory, antitumor, hepatoprotective, cardiovascular, antihelminthic and immunoregulatory activities [9]. The enormous medicinal use of these plants is mainly accounted to the presence of cucurbitacins [9].

*C. prophetarum,* belonging to the family Cucurbitaceae and genus *Cucumis,* is used by traditional healers in different part of the world to treat various ailments. In Saudi folk medicine, the fruit of *C. prophetarum* is used for the treatment of liver disorders [10]. In southern and central part of Ethiopia, the roots of *C. prophetarum* is traditionally used for the treatment of lung disorders, heart failure, back pain, diarrhea, gonorrhea, skin infections, intestinal problems and cancer [12, 13]. Previous chemical studies of the fruits of *C. prophetarum* has led to the isolation of cucurbitacin (B, E, I, O, P and Q1), dihydrocucurbitacin (B, D and E), isocucurbitacin (B, D and E) and dihydroisocucurbitacin (D and E) [11]. Therefore, this plant drew attention mainly due to its immense use in Ethiopian folk medicine for the treatment of various life threatening diseases. Despite the use of this plant against wide array of diseases, there is no information that dwells on the chemical constituents, antibacterial and antioxidant studies of the roots of *C. prophetarum.* In view of diverse traditional uses, we report herein the antibacterial, antioxidant, ADMET and molecular docking analysis of the chemical constituents of the roots of *C. prophetarum.*

## Results and discussion

**Fig. 1:**
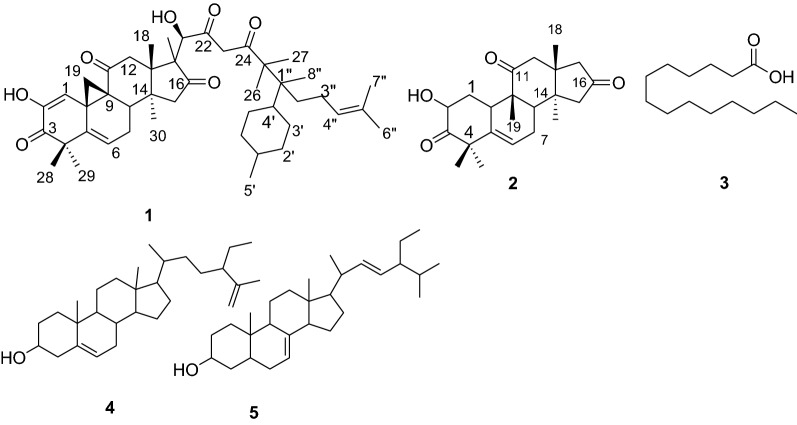
Compounds isolated from the roots of *C. prophetarum*

The root extracts of *C. prophetarum* after silica gel column chromatography furnished compounds 1–5, of which compounds 1 and 2 are novel compounds. The detailed characterizations of these compounds are presented below (Fig. 1).

Compound **1** (49 mg) was isolated as a yellow solid melting at 180–181 °C. TLC profile showed a spot at R_f_ 0.40 with *n*-hexane:EtOAc (4:1) as a mobile phase. The ^1^H-NMR (400 MHz, CDCl_3,_ Table 1) spectrum indicated the presence of three olefinic protons at *δ*_H_ 5.79 (1H, m, H-6), 5.95 (1H, s, H-1) and 5.97 (1H, m, H-4″). The spectrum also displayed proton signal at *δ*_H_ 3.92 (1H, brd s, H-21) which is evident for the presence of oxymethine proton (H-3). The presence of ten methyl signals are evident at *δ*_H_ 0.81 (3H, s, H-7″), 1.00 (3H, s, H-8″), 1.03 (3H, s, H-27), 1.10 (3H, s, H-26), 1.14 (3H, s, H-18), 1.25 (3H, s, H-30), 1.26 (6H, s, H-29), 1.34 (3H, s, H-6″), 1.38 (3H, s, H-20) and 3.56 (3H, s, H-28) which were accounted to methyl groups on quaternary carbons and one methyl proton signal at *δ*_H_ 1.27 (3H, *d*, *J* = 5.2 Hz, H-5′). The remaining proton signals were observed at *δ*_H_ 2.02 (2H, m, H-3″), 1.98 (2H, bro s, H-2″, H-19), 2.54 (2H, brd s, H-2″, H-19), 2.29 (2H, m, H-1′, H-8), 2.47 (2H, brd s, H-23), 2.39 (1H, s, H-15), 3.08 (1H, s, H-15) and 2.25 (1H, s, H-7).

**Table 1:** ^1^H & ^13^C-NMR spectroscopic data for cucurbitacins **1** and **2**

Compound 1			Compound 2		
Position	δ_H_	*δ*_C_	Position	δ_H_	*δ*_C_
1	5.95 (1H, brd s)	114.4	1	1.23 (1H, d, J = 4.4) 1.27 (1H, m)	36.1
2		144.6	2	4.43 (1H, dd, J = 6.0, 6.0)	71.5
3		198.5	3		212.8
4		47.6	4		50.3
5		136.9	5		140.5
6	5.79 (1H, m)	120.1	6	5.79 (1H, dd J = 2, 2)	119.8
7	2.07 (1H, m) 2.25 (1H, m)	50.2	7	2.43 (1H, brd s) 1.27 (1H, m)	24.3
8	2.29 (1H, m)	42.1	8	2.21 (1H, brd s)	42.9
9		50.3	9		45.0
10		49.8	10	2.79 (1H, d, J = 5.2)	33.9
11		215.9	11		215.9
12	2.07 (2H, m)	50.0	12	2.00 (1H, brd s) 2.07 (1H, brd s)	50.0
13		44.8	13		49.9
14		44.3	14		44.3
15	3.08 (1H, s) 2.39 (1H, s)	45.6	15	2.43 (1H, brd s) 3.17 (1H, s)	49.2
16		210.3	16		210.3
17		46.7	17	1.95 (1H, s) 2.53 (1H, s)	45.7
18	1.14 (3H, s)	19.1	18	1.23 (3H, s)	19.3
19	1.98 (1H, brd s) 2.54 (1H, brd s)	49.2	19	1.14 (3H, s)	20.0
20	1.38 (3H, s)	20.2	20	1.34 (3H, s)	21.2
21	3.92 (1H, brd s)	80.2	21	1.27 (3H, s)	29.2
22		210.0	22	1.00 s (3H, s)	24.3
23	2.47 (2H, brd s)	23.9			
24		211.0			
25		45.1			
26	1.10 (3H, s)	20.0			
27	1.03 (3H, s)	24.3			
28	3.56 (3H, s)	34.8			
29	1.26 (3H, s)	27.8			
30	1.25 (3H, s)	20.1			
1′	2.29 (1H, m)	43.3			
2′		23.9			
3′		24.2			
4′	2.79 (1H, m)	36.3			
5′	1.27 (3H, d, J = 5.2)	18.5			
1″		44.2			
2″	2.54 (1H, brd s) 1.98 (1H, d J = 3.2)	49.1			
3″	2.02 (2H, m)	45.9			
4″	5.97 (1H, m)	121.3			
5″		138.3			
6″	1.34 (3H s)	24.1			
7″	0.81 (3H, s)	20.9			
8″	1.00 (3H, s)	24.2			

The ^13^C-NMR (100 MHz, CDCl_3_, Table 1) spectral data with the aid of DEPT-135 revealed the presence of 43 carbon resonances corresponding to sixteen quaternary, seven methine, nine methylene and eleven methyls. Among them, five signals at *δ*_C_ 198.5 (C-3), 210.0 (C-22), 210.3 (C-16), 211.1 (C-24) and 215.9 (C-11) are easily attributable to carbonyls and six signals at *δ*_C_ 114.4 (C-1), 120.2 (C-6), 121.3 (C-4″), 136.9 (C-5), 138.3 (C-5″) and 144.6 (C-2) are due to olefinic carbons. The carbon signal observed at *δ*_C_ 80.2 (C-21) is obvious for carbon bearing oxygen (C-3). The spectrum displayed signals due methyl groups at *δ*_C_ 18.5 (C-5′), 19.1 (C-18), 20.0 (C-26), 20.1 (C-30), 20.2 (C-20), 20.9 (C-7″), 24.1 (C-6″), 24.2 (C-8″), 24.3 (C-27), 27.8 (C-29) and 34.8 (C-28). The signals due to methylene carbons are evident at *δ*_C_ 23.9 (C-23), 24.2 (C-3′), 23.9 (C-2′), 45.6 (C-15), 45.9 (C-3″), 49.1 (C-2″), 49.2 (C-19), 50.0 (C-12) and 50.2 (C-7). The remaining carbon signals were displayed at *δ*_C_ 36.3 (C-4′), 42.1 (C-8), 43.3 (C-1′), 44.2 (C-1″), 44.3 (C-14), 44.8 (C-13), 45.1 (C-25), 46.7 (C-17), 47.6 (C-4), 49.8 (C-10) and 50.3 (C-9). The above spectral features displayed by compound **1** are thus characteristic attributes of a cucurbitane type skeleton [14] with fifteen more carbons.

The COSY spectrum showed that the proton signals at *δ*_H_ 5.79 (H-6) and 5.97 (H-4″) correlated with protons at *δ*_H_ 2.07 (H-7) and methylene signal at *δ*_H_ 2.02 (H-3″), respectively. The HSQC spectrum showed correlation between the olefinic proton signals at *δ*_H_ 5.79 (H-6), 5.95 (H-1) and 5.97 (H-4″) with the olefinic carbons at *δ*_C_ 120.1 (C-6), 114.4 (C-1) and 121.3 (C-4″), respectively. The proton at *δ*_H_ 3.92 (H-21) correlated with the carbon at *δ*_C_ 80.2 (C-21). The spectrum also displayed correlation of proton signals at *δ*_H_ 3.56 (H-28), 3.08 (H-15) and 2.39 (H-15), 2.79 (H-4′), 2.29 (H-8,1′), 2.07 (H-7) and 2.25 (H-7), 2.07 (H-12), 1.26 (H-29), 1.38 (H-20), 1.34 (H-6″), 1.24 (H-30), 1.10 (H-26), 1.04 (H-27), 1.00 (H-8″), 0.81 (H-7″) with carbon signals at *δ*_C_34.9 (C-28), 45.6 (C-15), 36.3 (C-4′), 42.2 (C-8) and 43.3 (C-1′), 50.2 (C-7), 50.0 (C-12), 27.8 (C-29), 20.2 (C-20), 24.1 (C-6″), 20.1 (C-30), 20.0 (C-26), 24.3 (C-27), 24.2 (C-8″) and 20.9 (C-7″), respectively.

**Fig. 2:**
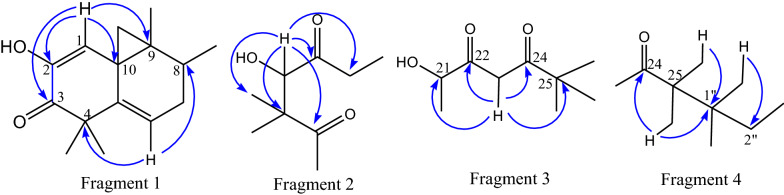
Structures of fragments 1–4 with important HMBC correlations

In the HMBC spectrum, the proton signal at *δ*_H_ 5.95 (H-1) had correlation with carbonyl carbon at *δ*_C_ 198.5 (C-3), two olefinic carbons at *δ*_C_ 144.6 (C-2) and 136.5 (C-5) and two quaternary carbons at *δ*_C_ 49.8 (C-10) and 50.3 (C-9) (Fig. 1). Long range correlation of proton signal at *δ*_H_ 5.79 (H-6) with signal at *δ*_C_ 47.6 (C-4) and *δ*_C_ 42.2 (C-8) (fragment 1, Fig. 2). The proton signal at *δ*_H_ 3.92 (H-21) showed strong long range correlations with two carbonyl carbons at *δ*_C_ 210.0 (C-22) and 210.3 (C-16), quaternary carbon at *δ*_C_ 46.7 (C-17), methylene carbons at *δ*_C_ 23.9 (C-23) and 20.2 (C-20) (fragment 2). Correlation was observed between the proton signal at *δ*_H_ 2.47 (H-23) with carbonyl signals at *δ*_C_ 210.0 (C-22) and 211.0 (C-24), quaternary carbon signal at *δ*_C_ 45.1 (C-25) and hydroxy bearing carbon signal at *δ*_C_ 80.2 (C-21) (fragment 3, Fig. 2). The proton signal at *δ*_H_ 1.10 (H-26) showed correlation with a carbonyl carbon signal at *δ*_C_ 211.0 (C-24) and proton signals at *δ*_H_ 1.00 (H-8″) and 1.10 (H-26) with quaternary carbon signal at *δ*_C_ 44.2 (C-1″) led us to fragment 4 (Fig. 2).

Careful inspection of literature reported revealed that all naturally occurring cucurbitacins have identical configuration at C-8, C-9, C-10, C-13 and C-14. This is because all of such compounds are biogenetically originated from the protosterylcation as precursor [15]. Based on the above spectral data, cucurbitacin **1** is found to be novel compound with its structure depicted in Fig. 1.

Compound **2** was isolated as a pale yellow solid melting at 118–120 °C. TLC profile showed a single spot at R_f_ 0.5 with *n*-hexane:EtOAc (1:1) as eluent. The ^1^H-NMR spectrum (400 MHz, CDCl_3,_ Table 1) displayed one olefinic proton signal at *δ*_H_ 5.79 (1H, dd, *J* = 2, 2 Hz, H-6). The spectrum also showed the presence of one oxymethine proton at *δ*_H_ 4.43 (1H, dd, *J* = 6.0, 6.0 Hz, H-2). The proton signal appearing at *δ*_H_ 1.00 (3H, s, H-22), 1.14 (3H, s, H-19), 1.23 (3H, s, H-18), 1.27 (3H, s, H-21) and 1.34 (3H, s, H-20) were accounted for the presence of five methyl groups on quaternary carbon. The spectrum also revealed the presence of proton signals at *δ*_H_ 1.23 (1H, *d*, *J* = 4.4, H-1), 1.27 (2H, m, H-1, H-7), 1.95 (1H, s, H-17), 2.00 (1H, brd s, H-12), 2.07 (1H, brd s, H-12), 2.21 (1H, brd s, H-8), 2.43 (2H, brd s, H-7, H-15), 2.53 (1H, s, H-17), 2.79 (1H, d, *J* = 5.2, H-10), 3.17 (1H, s, H-15).

The ^13^C-NMR spectrum (100 MHz, CDCl_3_, Table 1) with the aid of DEPT-135 revealed the presence of 22 carbon signals of which eight quaternary, four methine, five methylene and five methyl groups are clearly evident. Three carbon signals appearing at *δ*_C_ 210.3 (C-16), 212.8 (C-3) and 215.9 (C-11) were diagnostic for the presence of three carbonyl groups. The carbon resonances appearing at *δ*_C_ 140.5 (C-5) and 119.8 (C-6) were characteristics of olefinic carbons of which the former is quaternary olefinic carbon. One oxymethine carbon signal is evident at *δ*_C_ 71.5 (C-2). The other signal due to methine carbons were observed at *δ*_C_ 42.9 (C-8) and 33.9 (C-10). The carbon signals displayed at *δ*_C_ 36.1 (C-1), 24.3 (C-7), 50.0 (C-12), 49.2 (C-15) and 45.7 (C-17) were due to methylene carbons. The presences of methyl signals were apparent at *δ*_C_ 24.3 (C-22), 29.2 (C-21), 21.2 (C-20), 20.0 (C-19) and 19.3 (C-18).The remaining carbon resonances at *δ*_C_ 50.3 (C-4), 45.0 (C-9), 49.9 (C-13) and 44.3 (C-14) are due to quaternary carbons.

The COSY spectrum revealed that proton signal at *δ*_H_ 5.79 (H-6) and 4.43 (H-2) correlated with diastereotopic proton signals at *δ*_H_1.27, 2.43 (H-7) and *δ*_H_ 1.27, 1.23 (H-1), respectively. The HSQC spectrum revealed that the proton signal at *δ*_H_ 5.79 (H-6) and *δ*_H_ 4.43 (H-2) correlated with carbons at *δ*_C_ 119.8 (C-6) and *δ*_C_ 71.5 (C-2), respectively. HSQC spectrum also displayed correlation between proton signals at *δ*_H_ 1.00 (H-22), 1.14 (H-19), 1.23 (H-18), 1.27 (H-21) and 1.34 (H-20) with the carbon signals at 24.2 (C-22), 20.0 (C-19), 19.2 (C-18), 29.2 (C-21) and 21.2 (C-20), respectively. Diastereotopic protons at *δ*_H_ 1.27, 2.43 (H-7) and *δ*_H_ 1.27, 1.23 (H-1) correlated with carbon signals at *δ*_C_ 24.3 (C-7) and *δ*_C_ 36.1 (C-1), respectively.

The HMBC spectrum showed that proton signal at *δ*_H_ 5.79 (H-6) has strong long range correlation with two quaternary carbons at *δ*_C_ 140.5 (C-5) and 50.3 (C-4), and two methine signals at *δ*_C_ 33.9 (C-10) and 42.9 (C-8). The HMBC spectrum also showed long range correlation of proton signal at *δ*_H_ 4.43 (H-2) with carbonyl carbon at *δ*_C_ 212.8 (C-3) and methylene carbon at signal *δ*_C_ 36.1 (C-1). Long range correlations between proton signal at *δ*_H_ 2.00 (H-12) with carbonyl at *δ*_C_ 215.9 (C-11), methyl carbon at *δ*_C_ 19.3 (C-18) and three quaternary carbon signals at *δ*_C_ 49.9 (C-13), 45.0 (C-9) and 44.3 (C-14) were also clearly evident. Methylene proton signal at *δ*_H_ 2.43 (H-15) showed correlation with the carbon signals at *δ*_C_ 210.3 (C-16), 49.9 (C-13) and 44.3 (C-14) and 24.3 (C-22) which helped unequivocal assignment of carbonyl carbon appearing at *δ*_C_ 210.3 (C-16) to C-16 position of ring D. Thus, based on the above spectral data cucurbitacin 2 (Fig. 1) was suggested which is found to be novel compound.

Compound **3** (25 mg) was isolated as a white solid from the *n*-hexane extract of the roots of *C. prophetarum*. It melted at 49–50 °C. TLC profile showed a spot at R_f_ 0.6 with *n*-hexane: EtOAc (9:1) as eluent. The ^1^H-NMR spectral data (400 MHz, CDCl_3_) showed signal due to terminal methyl protons at *δ*_H_ 0.90 (3H, t, *J* = 6.1 Hz). The spectrum also displayed methylene signals at *δ*_H_ 2.36 (2H, t) and 1.64 (2H, m) where the former suggests a methylene protons α to a carboxyl group. The proton signal centered at *δ*_H_ 1.27 (20H, brd s) is diagnostic for the presence of many overlapping methylene groups. The ^13^C-NMR spectrum (100 MHz, CDCl_3_) with the help of DEPT-135 revealed the presence of twelve well resolved carbon resonances of which one carbonyl carbon at *δ*c 179.2, one methyl at *δ*c 14.2 and the remaining ten carbons due to methylenes at *δ*c 22.7, 24.7, 29.0, 29.3, 29.4, 29.5, 29.6, 29.7, 31.9 and 34. Thus, based on the above spectral data, compound **3** was found to be identical with myristic acid (Fig. 1).

Compound **4** (67 mg) was isolated as pale yellow solid from the *n*-hexane extract of the roots of *C. prophetarum.* It melted at 140–141 °C. The TLC profile showed a single spot at R_f_ 0.6 with *n*-hexane: EtOAc (4:1) as mobile phase. The ^1^H-NMR (400 MHz, CDCl_3_) and ^13^C-NMR (100 MHz, CDCl_3_) spectral data comparison revealed good agreement with that of (24S)-24-ethylcholesta-5,25-diene-3-one [16] except the C-3 carbonyl is reduced to alcohol in case of compound 4. This was confirmed by the appearance of a siganl at *δ*_H_ 3.66 (1H, m) and *δ*c 78.8 in the ^1^H and ^13^C-NMR spectrum, respectively, and disappearance of a signal at *δ*c 210 in the ^13^C-NMR spectrum which is typical signal observed in spectral data of (24S)-24-ethylcholesta-5,25-diene-3-one.

Compound **5** (158 mg) was isolated as a white solid from *n*-hexane extract of the roots of *C. prophetarum* and *C. ficifolius*. It melted at 172–173^▫^C (Lit. 169–171 °C) [17]. TLC profile showed a single spot at R_f_ 0.46 with *n*-hexane: EtOAc (7:3) as eluent. The ^1^H-NMR (400 MHz, CDCl_3_) spectrum showed three olefinic proton signals at *δ*_H_ 5.11 (1H, m), 5.16 (1H, dd, *J* = 2,4) and 5.17 (1H, brd s). The proton signal at *δ*_H_ 3.61 (1H, m) suggest the presence of oxymethine proton. The spectrum also displayed signals due to six methyl groups at *δ*_H_ 0.56 (3H, s), 0.80 (3H, s), 0.81 (3H, m), 0.83 (3H, d, *J* = 1.6), 0.85 (3H) and 1.03 (3H, s). The remaining signals are accounted to methylene and methine protons. The ^13^C-NMR (100 MHz, CDCl_3_) spectrum with the aid of DEPT-135 revealed the presence of well resolved twenty nine carbon resonances of which four olefinic carbons signals were observed at *δ*_C_ 117.4, 129.4, 138.2 and 139.6. The earlier signal is diagnostic for *Δ*^7^ sterol which excludes the possibility of *Δ*^5^ sterols [18]. Thus, the NMR spectral data of compound **5** was found to be in good agreement with that of *α*-spinasterol (**5**) reported in literature [17–19].

### Antibacterial activity of the extracts and isolated compounds

The antibacterial activity of the extracts and isolated compounds of roots of *C. prophetarum* were investigated using agar well diffusion methods against two Gram positive (*S. aureus* and *B. subtilis*) and two Gram negative (*E. coli* and *S. thyphimurium*) bacteria. The results were presented in Table 2.

**Table 2:** Antibacterial activities of extracts and isolated compounds from the root extracts of *C. prophetarum*

Samples	Conc (mg/mL)	Diameter Zone of Inhibition (DZI) in mm			
		Gram negative bacteria			Gram positive bacteria
		*E. coli*	*S. thyphimurium*	*S. aureus*	*B. subtilis*
**HECP**	5.0	15.0 ± 1.41	12.0 ± 1.41	11.6 ± 0.48	12.6 ± 1.15
**MECP**	5.0	14.6 ± 1.70	12.6 ± 2.05	11.0 ± 0.01	12.3 ± 0.47
**1**	2.5	12.3 ± 0.6	12.1 ± 0.01	10.6 ± 0.47	12.3 ± 0.08
	5.0	12.6 ± 0.94	13.1 ± 0.81	10.6 ± 0.47	12.6 ± 0.47
	7.5	13.6 ± 0.94	13.2 ± 1.41	11.3 ± 0.94	12.6 ± 0.47
**2**	2.5	12.0 ± 0.81	12.6 ± 0.47	11.0 ± 1.41	12.3 ± 0.47
	5.0	12.1 ± 1.63	12.6 ± 0.47	12.1 ± 1.41	12.6 ± 0.47
	7.5	12.3 ± 1.25	13.1 ± 1.41	12.1 ± 2.16	13.3 ± 0.54
**3**	5.0	11.1 ± 0.81	10.6 ± 1.24	9.0 ± 0.21	10.3 ± 0.47
**4**	5.0	12.0 ± 0.81	11.6 ± 0.94	10.6 ± 0.47	11.6 ± 1.25
**5**	2.5	11.1 ± 1.29	10.6 ± 1.70	10.6 ± 0.94	9.1 ± 0.03
	5.0	13.0 ± 0.10	12.3 ± 0.94	11.3 ± 0.94	10.2 ± 0.06
	7.5	13.2 ± 0.81	12.6 ± 1.25	12.3 ± 1.25	10.4 ± 1.41
**Ciprofloxacin**	5.0	18.7 ± 1.63	20.0 ± 1.63	19.0 ± 2.08	20.7 ± 0.94

Results are mean ± SD of triplicate experiments

HECP: hexane extract of C. *prophetarum*; MECP: MeOH extract of C. *prophetarum*

Various literature reports have shown that plant extracts and their constituents have been explored for their biological activities against various strains of bacterial pathogens [20]. In view of this, the extracts and isolated compounds from the roots of *C. prophetarum* showed differences in the inhibition of growth of bacteria against different strains of microorganisms. The absolute values of the DZI varied from 9.0 ± 0.21 mm to 15.0 ± 1.41 mm. The hexane and methanol extract of the root of *C. prophetarum* exhibited a very high inhibition diameter with magnitude of 15.0 and 14.6 against *E. coli*, respectively. The result is comparable with chloramphenicol used as positive control for the same strain. The antibacterial activity of the extracts of the roots of *C. prophetarum* were also compared with literature reported for sister species including *C. sativus* and *C. melo.* Interestngly better antibacterial activity was obatined in this work even at lower concentration against similar bacterial strain compared with the extract of the flower of *C. sativus* and seeds of *C. melo* [21, 22].

Quantitative evaluation suggests that all the compounds tested displayed broad range of antibacterial activity with DZI ranging from medium to very high. The best result was achieved by compound 1, inhibition zone of 13.6 ± 0.94 mm, against *E. coli.* This is close to the activity displayed by ciprofloxacin. Furthermore, the inhibition zone of all the compounds in the present study increased with increasing concentration in a dose dependent manner. As observed from Table 2, the extracts displayed a slight better antibacterial activity compared with the isolated compounds. The better activity of the extracts might be due to the synergistic interactions of several phytochemicals present in the extract. The wide zone of inhibition of the extracts and isolated compounds of the roots extract of *C. prophetarum* showed that it had great potential as a remedy for infections/diseases caused by bacterial pathogens. Furthermore the results obtained in the present work corroborate the traditional use of the roots of *C. prophetarum* against bacteria.

### Molecular docking binding analysis of isolated compounds (1–5) docked against *E.coli* DNA gyraseB (PDB ID 6F86)

DNA gyrase plays a crucial role in bacterial survival and therefore bacterial DNA gyrase has been a well explored antibacterial drug target [23–26]. Hence, in the present investigation, the molecular docking analysis of the isolated compounds was carried out to investigate their binding pattern with DNA gyrase and compared with the clinically approved drug ciprofloxacin. The isolated compounds (**1–5**) were found to have minimum binding energy ranging from – 6.1 to – 6.9 kcal/mol (Table 3). The isolated compounds **2** and **3** shown comparable results (binding score and amino acid interactions) compared to the standard inhibitor ciprofloxacin. The binding affinity, H-bond and hydrophobic, pi-cation and Van der Waals interactions of ligands (**1–5**) were summarized in Table 3. The natural product compounds **1–5** isolated revealed moderate to better docking affinity (− 5.6 to − 6.9) within the binding pocket of *E.coli* DNA gyrase B. All the isolated compounds shown the residual amino acids interactions with Ala-47, Glu-50, Gly-77, Ile-78, Pro-79, Ile-94, Thr-165 (Hydrophobic) and Asp-73, Arg-76, Asn-46 (Hydrogen bond) similar to ciprofloxacin within the binding region. Compound **1** forms hydrogen bond interaction with Glu-50 in the active site of the target protein with the least binding affinity − 6.4 kcal/mol. Compound **4** has the least binding affinity − 5.6 kcal/mol with one hydrogen bond (Asp-73) and hydrophobic interaction (Ile-78) within the binding pocket. The compounds **4** and **5** each formed a hydrogen bond interaction with Asp-73 within the binding region with least binding affinity − 5.6 and − 6.5 kcal/mol, respectively. Compound **2** formed hydrogen bond interaction (Gly-77 and Thr-165) and residual Van dar Waals interaction with residual amino acids Asp-73, Ile-78, and Ile-94. The Molecular docking study of these compounds displayed comparable docking score (except 4) within binding pocket toward *E. coli* DNA gyrase B (6F86). Overall, in silico docking analysis of the compounds (**1–5**) binding affinity are matching with in vitro analysis against *E.coli* and *S. aureus*, in addition to that all the compounds shown comparable binding affinity and residual interaction of ciprofloxacin. Based on the results, all the compounds might be the better anti-bacterial agent in this investigation. The binding affinity, H-bond and residual amino acid interactions of six compounds are summarized in Table 3.

**Table 3:** Molecular docking value of isolated compounds (**1–5**) against DNA gyrase B (PDB ID: 6F86)

Ligands	Binding Affinity (kcal/mol)	H-bond	Residual interactions	
			Hydrophobic/Pi-Cation	Van dar Waals
**Compound 1**	− 6.4	Glu-50	Pro-79	Asp-73, Asn-46, Ala-47, Arg-76, Gly-77, Ile-78, Gly-81, Ile-94, Arg-136, Thr-165
**Compound 2**	− 6.7	Gly-77, Thr-165	Glu-50	Asp-73, Ile-78, Ile-94
**Compound 3**	− 6.9	Gly-77, Asn-46, Thr-165	–	Asp-73, Ala-47, Asp-49, Glu-50, Gly-75, Arg-76, Ile-78, Pro-79, Ile-94, Arg-136
**Compound 4**	− 5.6	Asp-73	Ile-78	Asn-46, Ala-47, Glu-50, Arg-76, Gly-77, Pro-79, Ile-94, Thr-165
**Compound 5**	− 6.5	Asp-73	Ile-78, Pro-79, Ile-94	Asn-46, Ala-47, Glu-50, Arg-76, Gly-77, Arg-136, Thr-165,
**Ciprofloxacin**	− 6.9	Asp-73, Asn-46, Arg-76	Ile-78, Ile-94, Glu-50, Gly-77	Ala-47, Pro-79, Thr-165

**Fig. 3:**
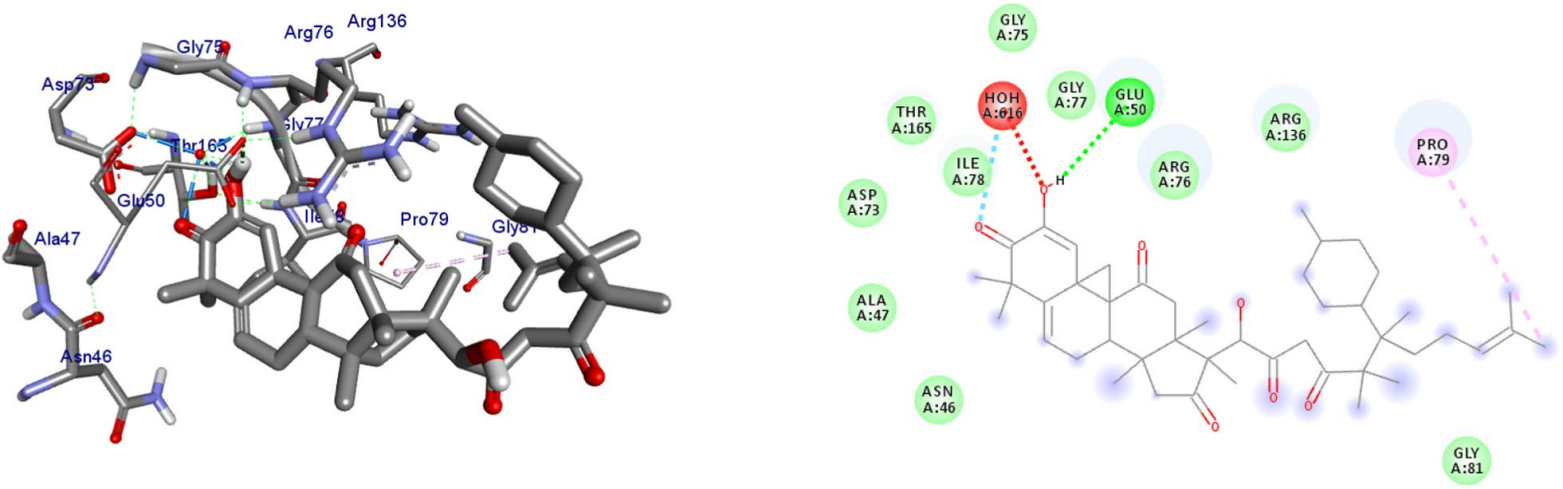
The 2D and 3D binding interactions of compound **1** against DNA gyrase B (PDB ID: 6F86)

**Fig. 4:**
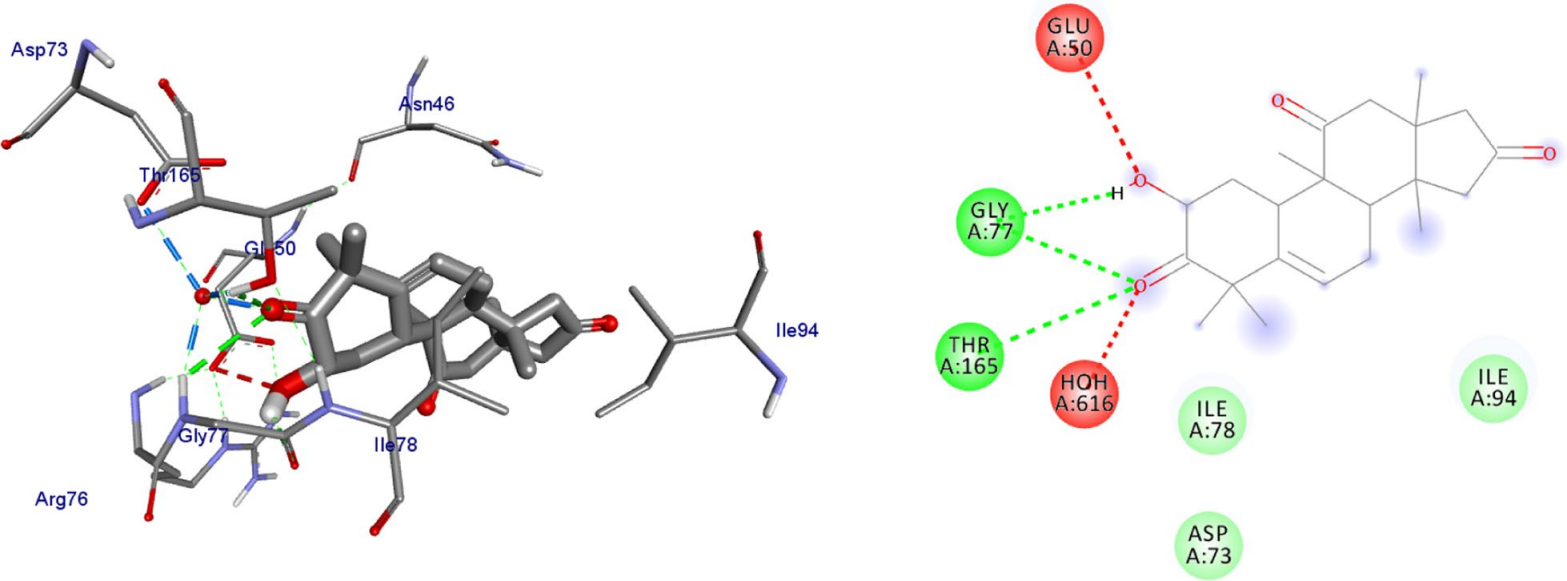
The 2D and 3D binding interactions of compound 2 against DNA gyrase B (PDB ID: 6F86)

**Fig. 5:**
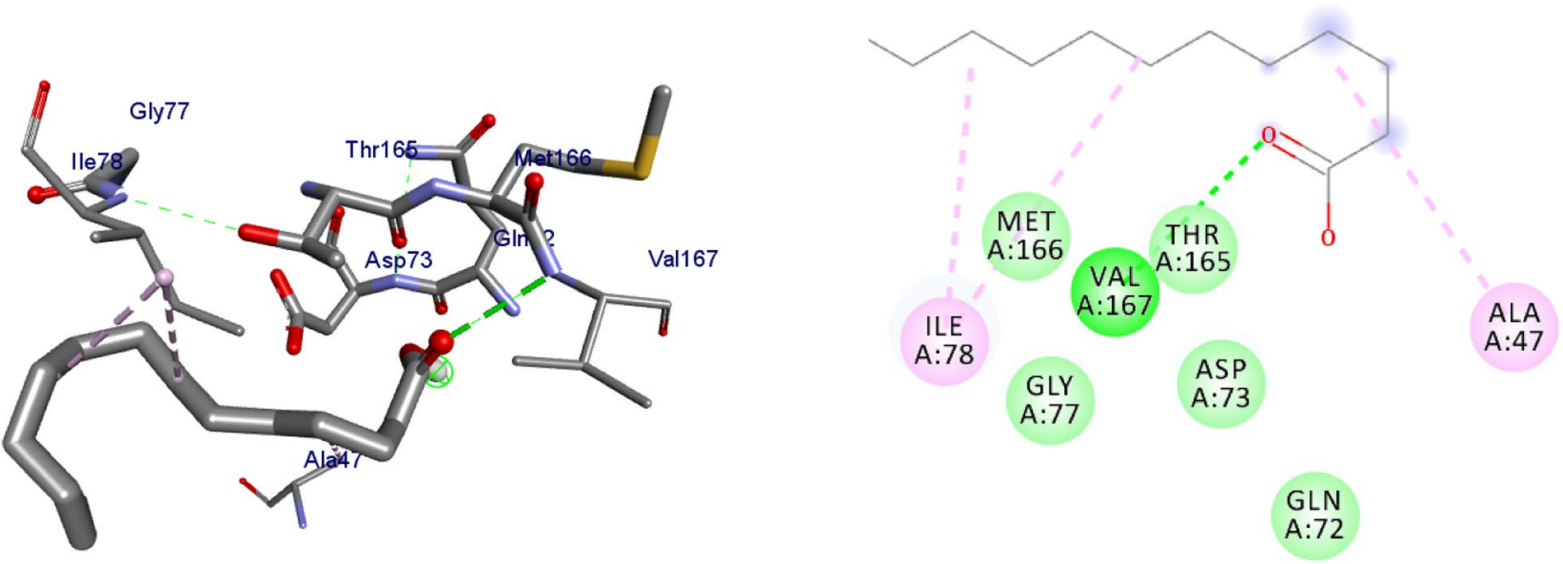
The 2D and 3D binding interactions of compound **3** against DNA gyrase B (PDB ID: 6F86)

**Fig. 6:**
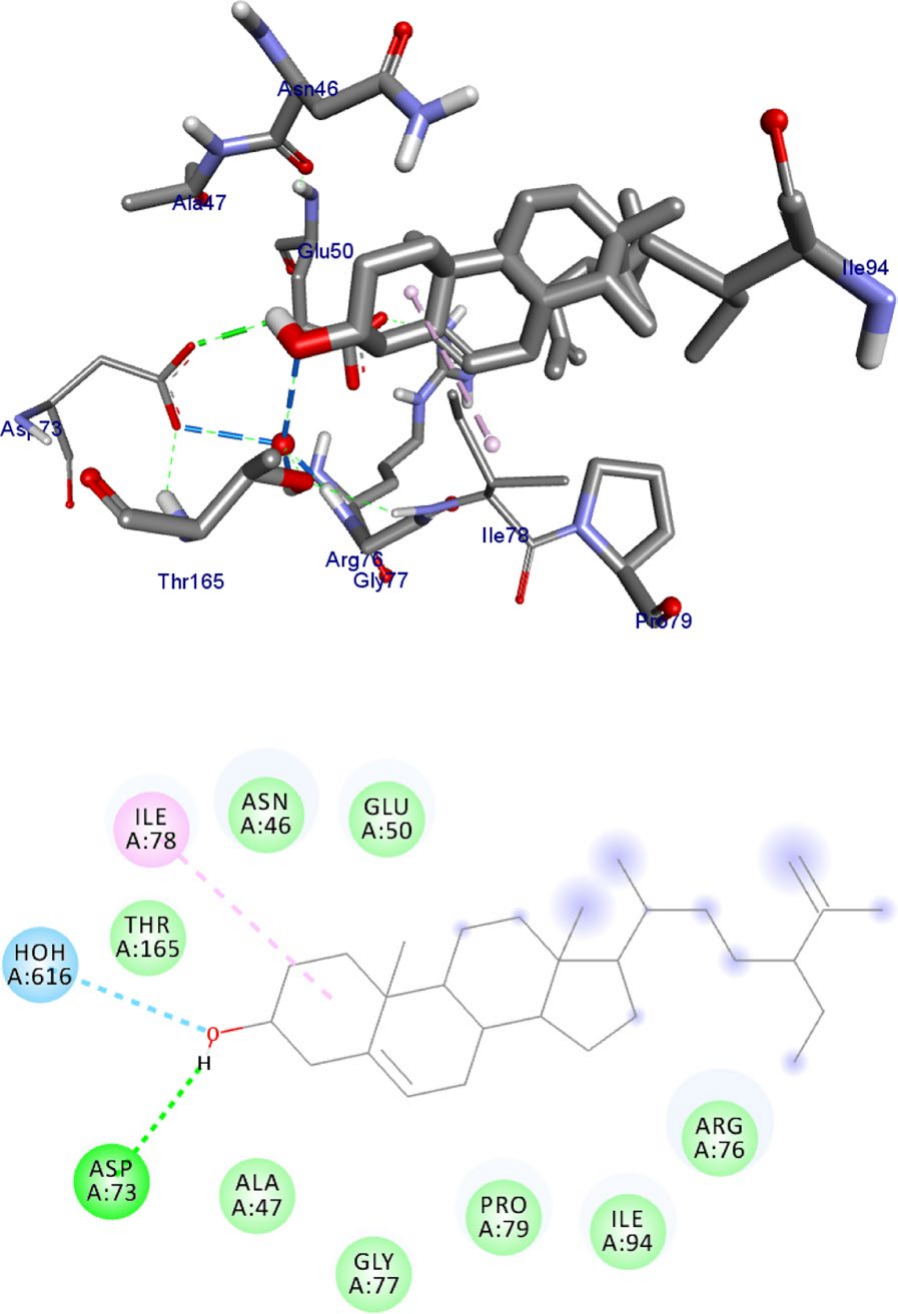
The 2D and 3D binding interactions of compound **4** against DNA gyrase B (PDB ID: 6F86)

**Fig. 7:**
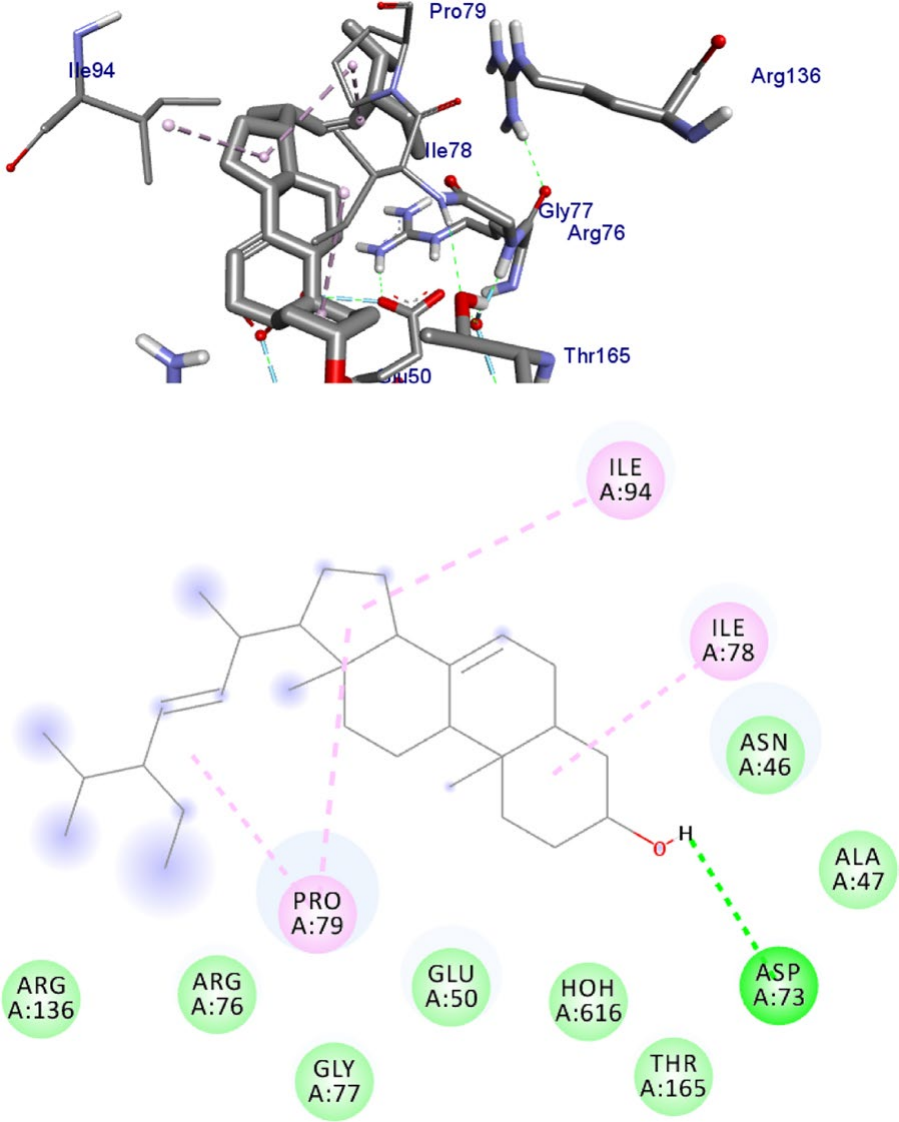
The 2D and 3D binding interactions of compound **5** against DNA gyrase B (PDB ID: 6F86)

**Fig. 8:**
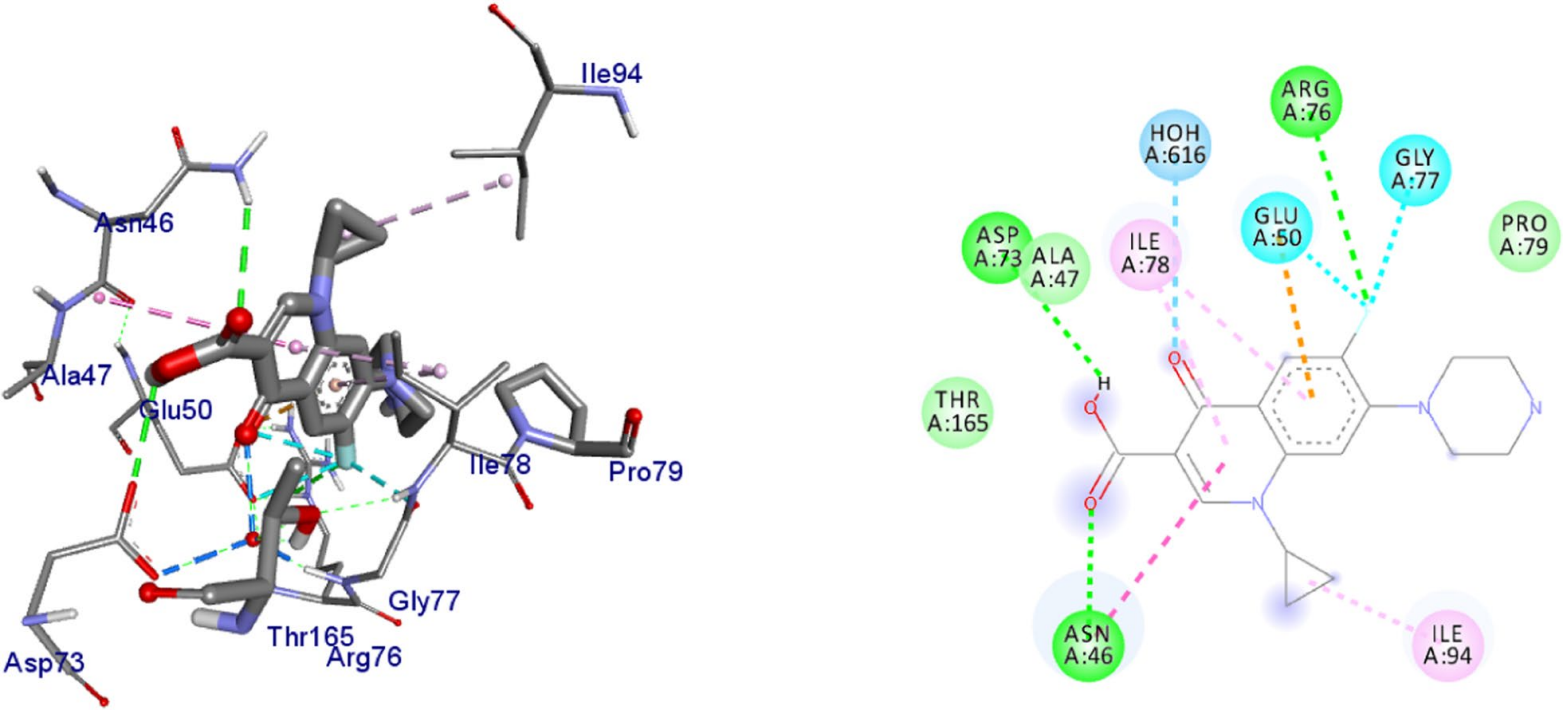
The 2D and 3D binding interactions of ciprofloxacin against DNA gyrase B (PDB ID: 6F86)

The binding interactions of compounds **1–5** and ciprofloxacin against DNA gyrase B are shown in Figs. 3, 4, 5, 6, 7, 8. Ribbon model shows the binding pocket structure of DNA gyrase B with the compounds. Hydrogen bond between compounds and amino acids are shown as green dash lines, hydrophobic interaction are shown as pink lines.

### Radical scavenging activity and anti-lipid peroxidation activities of extracts and isolated compounds

DPPH radical scavenging assay is a simple method for finding antioxidant activities of samples by recording absorbance at 517 nm [27]. The decrease in absorbance at 517 nm [28] in addition to the change in color of the DPPH from purple to yellow indicates the antioxidant activity of the samples. The results of the radical scavenging activity of the extracts and isolated compounds from the roots of *C. prophetarum* and *C. ficifolius* were summarized in Table 4.

**Table 4:** DPPH radical scavenging and anti-lipid peroxidation activities of extracts and constituents of the roots of *C. prophetarum*

Samples	%DPPH inhibition at				IC_50_	Anti-lipid peroxidation % inhibition
	100 (µg/mL)	50 (µg/mL)	25 (µg/mL)	12.5 (µg/mL)		
**HECP**	63.30 ± 0.04	56.10 ± 0.10	37.51 ± 0.03	26.61 ± 0.04	47.3	34 ± 0.05
**MECP**	70.41 ± 0.01	62.42 ± 0.09	52.62 ± 0.07	38.12 ± 0.10	28.9	53 ± 0.10
**1**	57.11 ± 0.10	38.50 ± 0.4	26.20 ± 0.05	14.43 ± 0.21	81.2	42 ± 0.09
**2**	56.22 ± 0.70	41.31 ± 0.5	29.92 ± 0.05	24.42 ± 0.14	80.2	44 ± 0.09
**3**	26.32 ± 0.03	22.62 ± 0.05	16.73 ± 0.08	10.24 ± 0.02	232.3	22 ± 0.09
**4**	26.20 ± 0.31	15.23 ± 0.9	10.23 ± 0.05	6.81 ± 0.08	208.7	16 ± 0.09
**5**	31.92 ± 0.27	24.71 ± 0.5	16.81 ± 0.50	10.82 ± 0.04	172.7	17 ± 0.09
**Ascorbic acid**	90.02 ± 0.02	76.60 ± 0.51	63.20 ± 0.15	50.10 ± 0.67	2.14	87 ± 0.02

Samples were reported as Mean ± SEM; Ascorbic acid was used as positive control

The extracts and isolated constituents of the roots of *C. prophetarum* reduce the stable DPPH radical to the yellow-colored diphenylpicrylhydrazicine indicating their potential as radical scavengers. As shown in Table 4, the DPPH radical scavenging activities of the extracts and isolated constituents of the roots of *C. prophetarum* increases with increasing concentrations. The methanol extract of the roots of *C. prophetarum* inhibited the DPPH radical by 70.4 at 100 µg/mL, respectively. At the same concentration standard ascorbic acid scavenged the DPPH radical by 90.0%. On the other hand, the hexane extracts of the roots of *C. prophetarum* displayed % inhibition of 63.3%. It was also noticed that all the isolated compounds exhibited lower percentage inhibition of the DPPH radical compared with the extracts at the same concentrations. This is likely accounted to the presence of phenols and flavonoids as minor constituents in the extracts of the roots of *C. prophetarum.* In agreement to this assertion is the formation a yellow orange color on treating the extracts of the roots of *C. prophetarum* with NaOH followed by HCl [29] which infers the presence of flavonoids. Moreover the extracts furnished a bluish green color on treatment with FeCl_3_ which infers for the presence of phenolics [30].

The extracts and isolated compounds were also evaluated for their anti-lipid peroxidation potential using ferric thiocyante method with the result presented in Table 4. As clearly seen in Table 4, the methanol extracts of the roots of *C. prophetarum* inhibit peroxide formation by 53%, demonstrating their potential in preventing the formation lipid peroxides. However, the isolated constituents exhibited lower ability of inhibiting peroxide formation compared with the extracts and the natural antioxidant. This is ascribed to the presence of flavonoids and phenols in extracts which were confirmed using alkaline and FeCl_3_ test respectively.

### Molecular docking binding analysis of isolated compounds (1–5) docked against human peroxidoxin 5 (PDB ID: 1HD2)

**Fig. 9:**
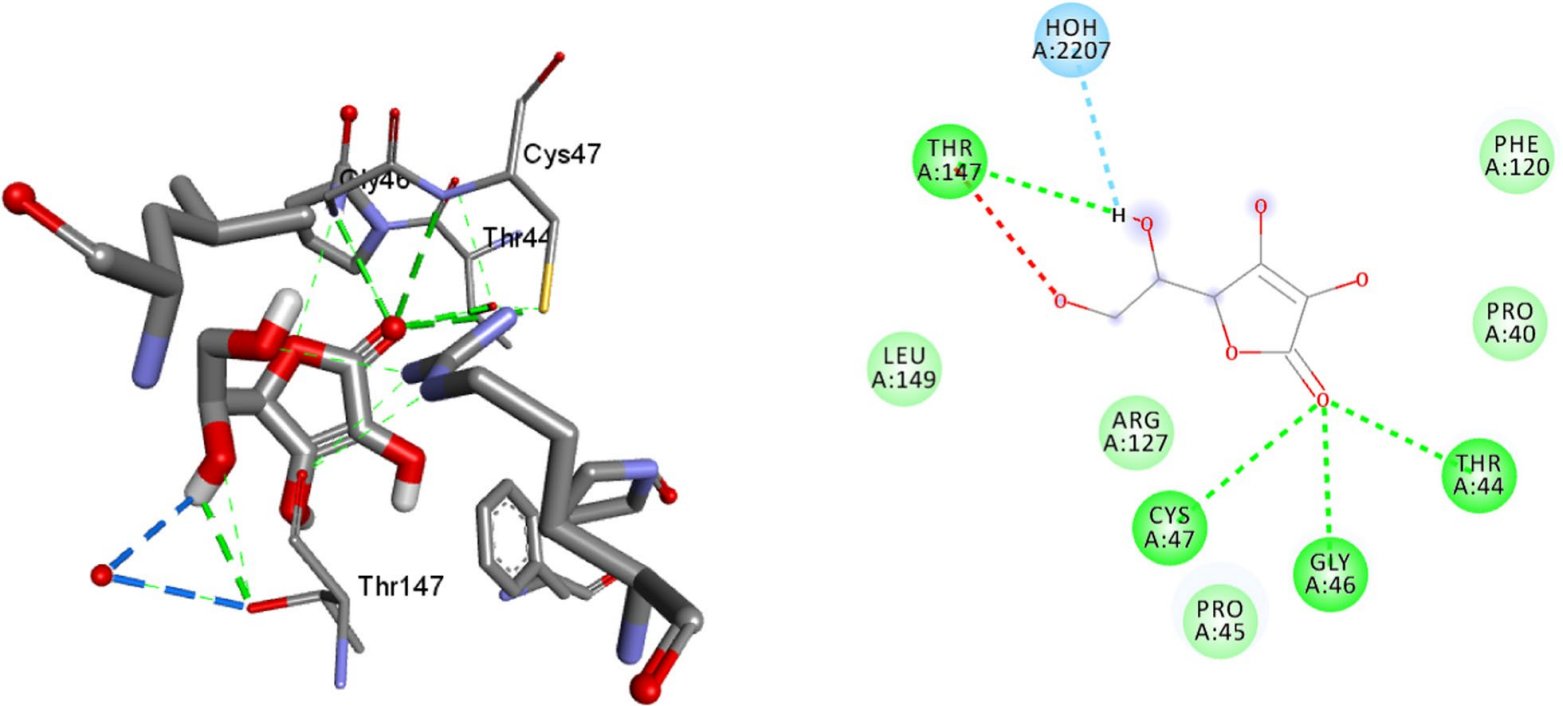
The 2D and 3D binding interactions of ascorbic acid against human peroxiredoxin 5 (PDB ID: 1HD2)

**Fig. 10:**
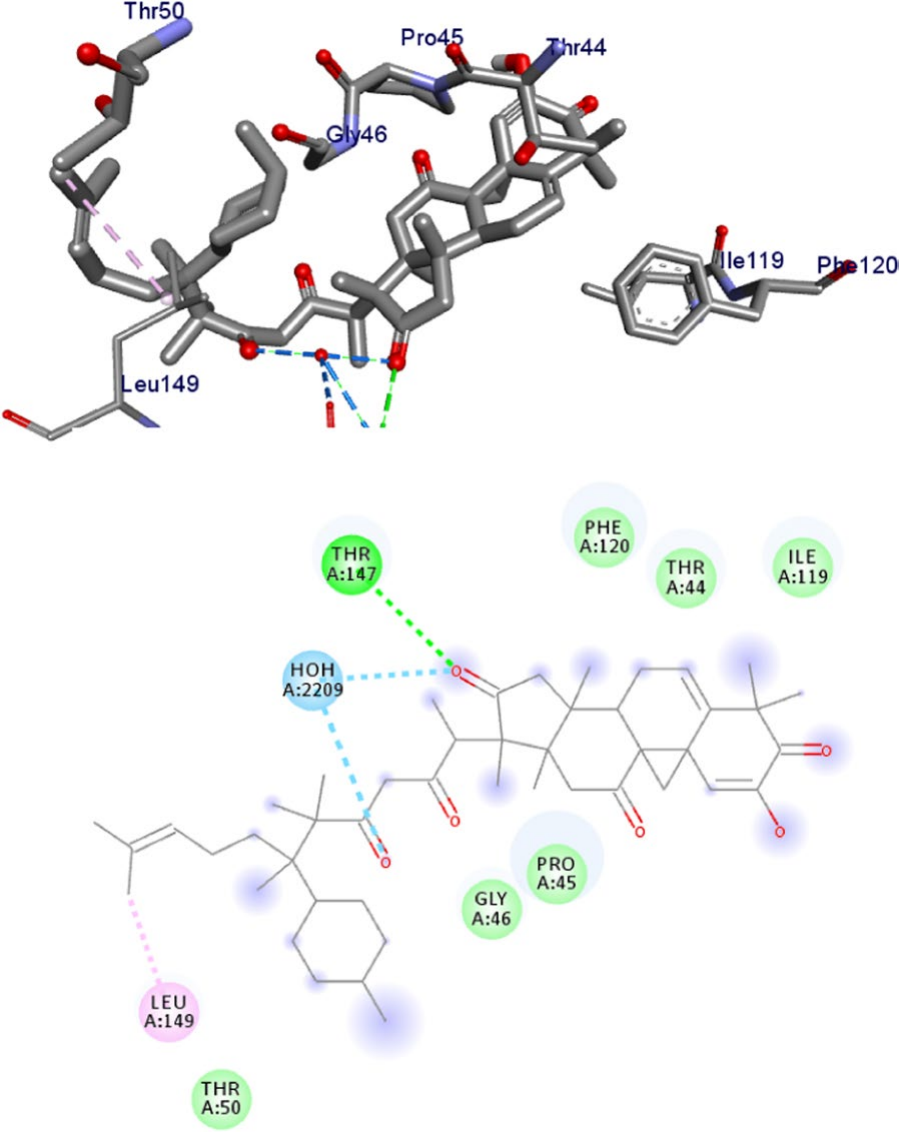
The 2D and 3D binding interactions of **1** against Human peroxiredoxin 5 (PDB ID: 1HD2)

**Fig. 11:**
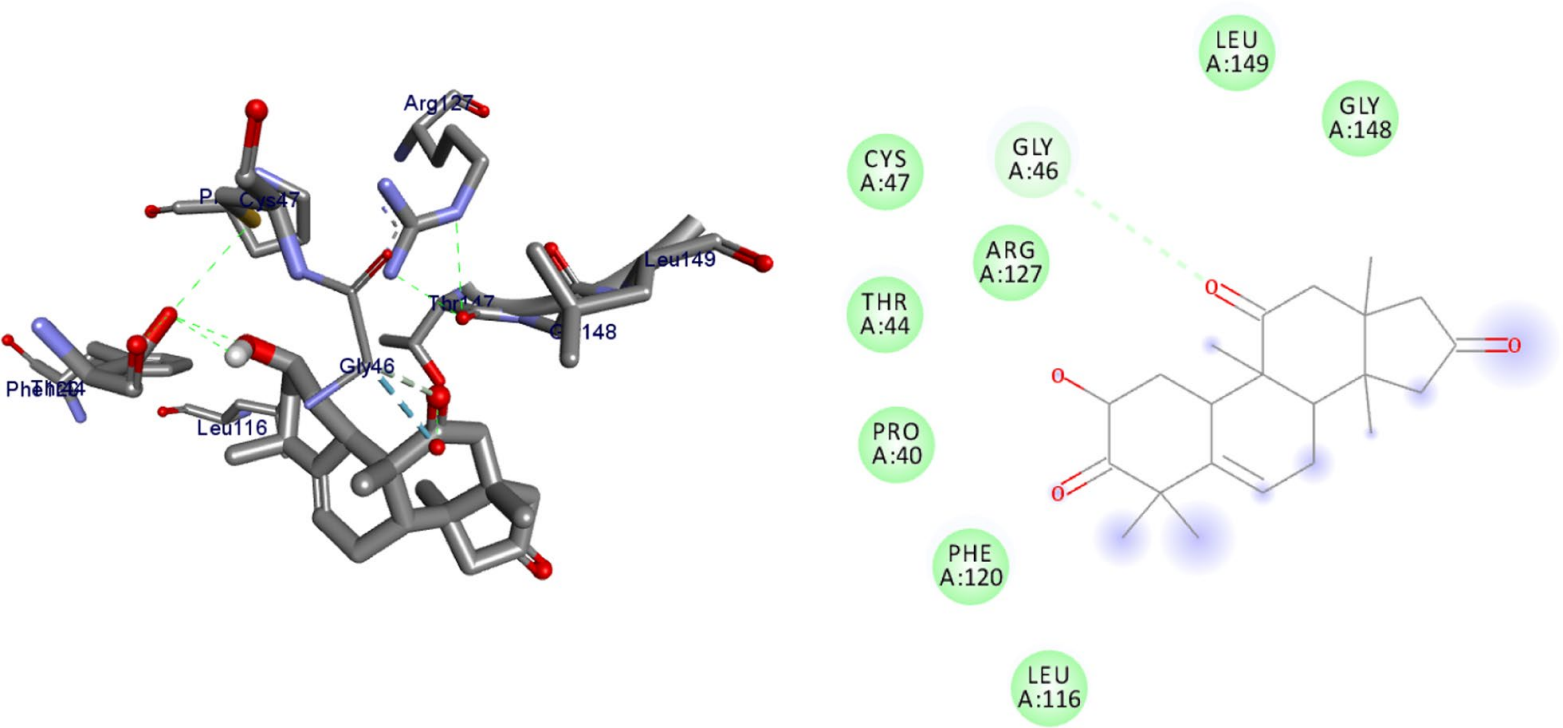
The 2D and 3D binding interactions of **2** against Human peroxiredoxin 5 (PDB ID: 1HD2)

**Fig. 12:**
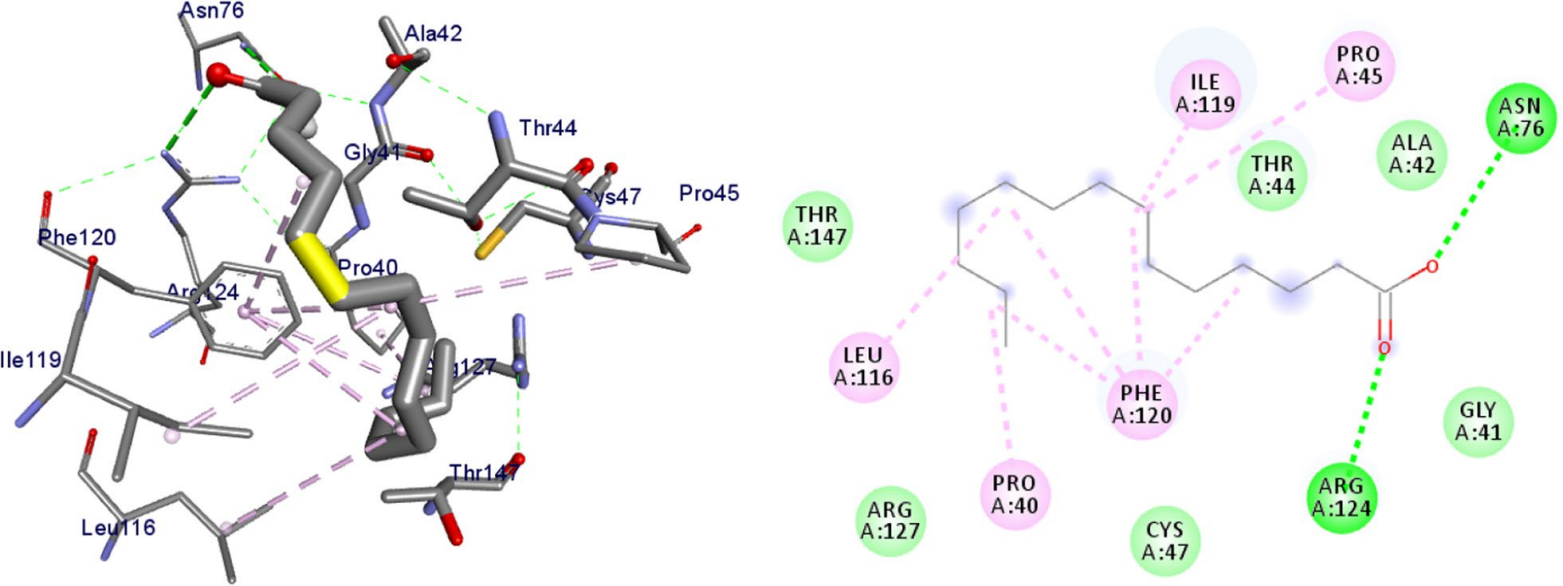
The 2D and 3D binding interactions of **3** against Human peroxiredoxin 5 (PDB ID: 1HD2)

**Fig. 13:**
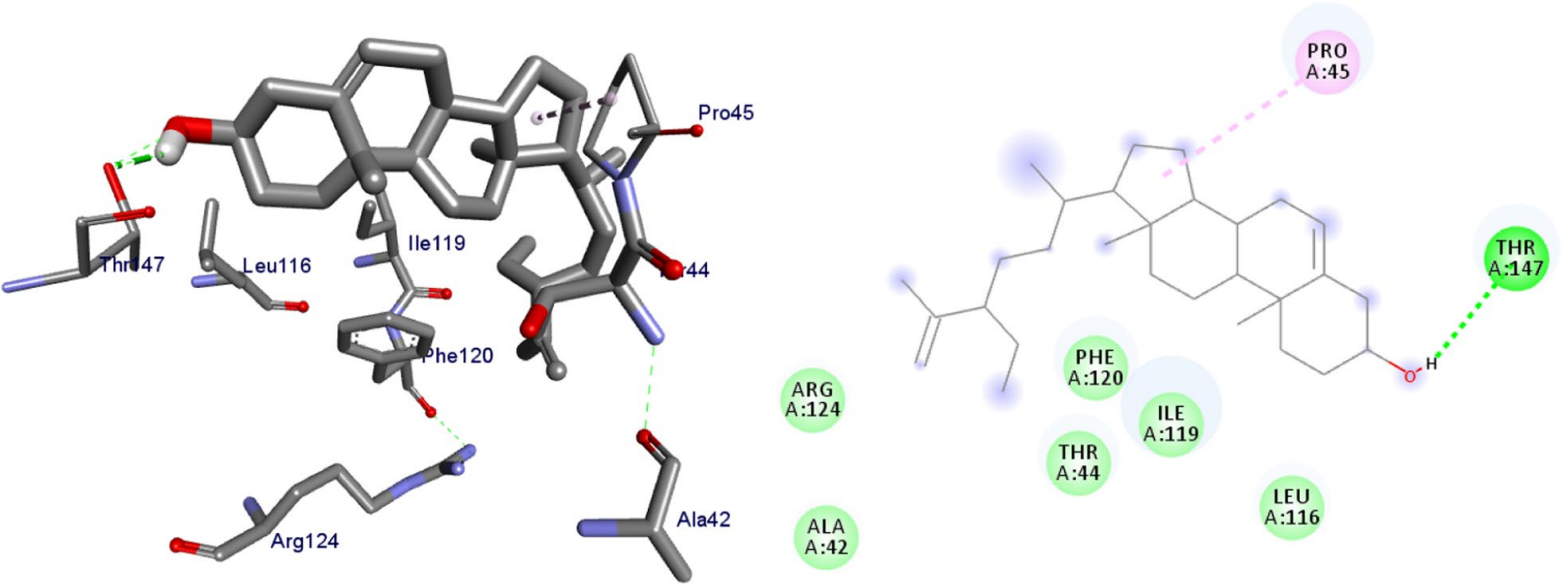
The 2D and 3D binding interactions of **4** against Human peroxiredoxin 5 (PDB ID: 1HD2)

**Fig. 14:**
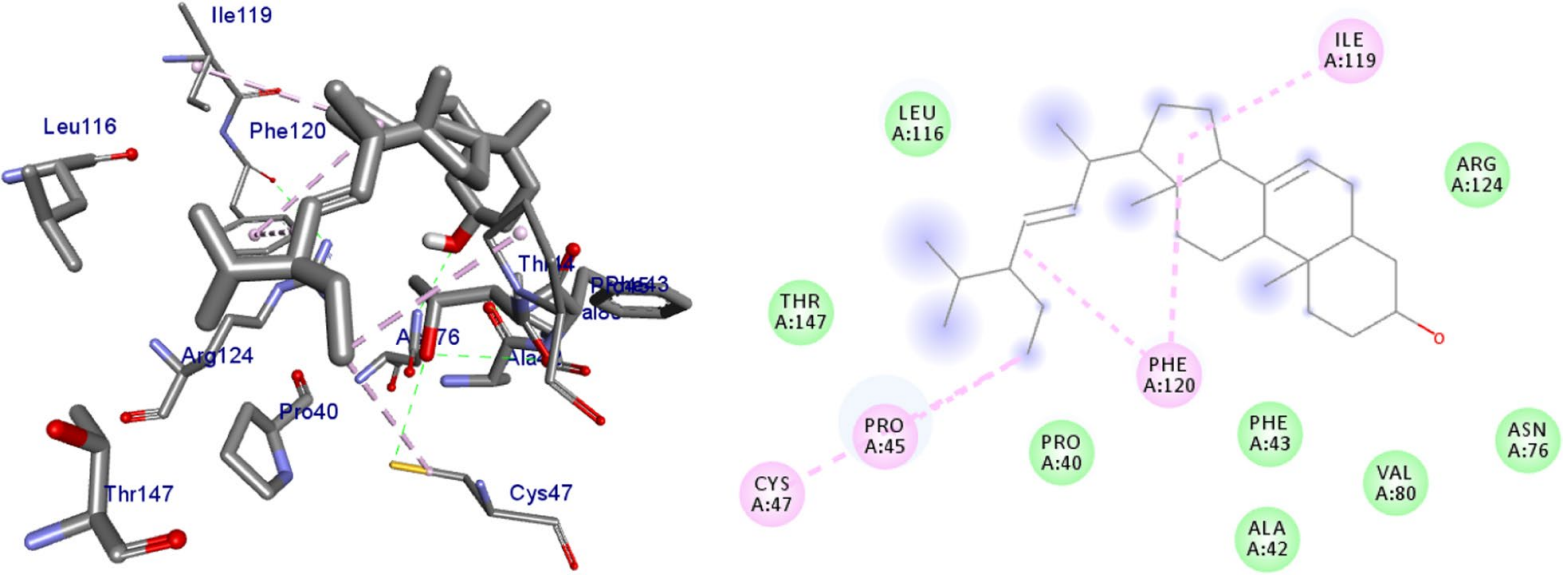
The 2D and 3D binding interactions of **5** against Human peroxiredoxin 5 (PDB ID: 1HD2)

The molecular docking analysis of the isolated compounds was carried out to investigate their binding pattern with human peroxidoxin (PDB ID: 1HD2) and compared with the natural antioxidant ascorbic acid. The isolated compounds (**1–5**) were found to have minimum binding energy ranging from − 4.5 to − 5.2 kcal/mol (Table 5). The binding affinity, H-bond and hydrophobic, pi-cation and Van der Waals interactions of ligands (**1–5**) were summarized in Table 5. The antioxidant analysis results of isolated natural product compounds **1–5** revealed moderate activity. The molecular docking analysis also shows the moderate to better docking affinity (− 4.5 to − 5.2 kcal/mol) within the binding pocket of human peroxidoxin 5. The key amino acid residues within the active sites of peroxidoxin **5** are Cys 47, Thr-44, Gly-46 and Thr-147. The compounds **1** and **2** formed hydrogen bond interaction with Thr-147 and Gly-46, respectively. The compound **5** formed a hydro (Gly-77 and Thr-165) and residual Van dar Waals interaction with residual amino acids Asp-73, Ile-78, and Ile-94. The Molecular docking study of these compounds displayed comparable docking score within binding pocket toward peroxidoxin 5. Overall, in silico docking analysis of the compounds (**1–5**) binding affinity are matching with antioxidant analysis, in addition to that all the compounds shown comparable binding affinity and residual interaction of ascorbic acid. The results show that compounds **1**, **2**, and **5** are potential antioxidants compared to other isolated compounds. The binding affinity, H-bond and residual amino acid interactions of six compounds are summarized in Table 5 and the binding interactions of them against peroxidoxin 5 are shown in Figs. 9, 10, 11, 12, 13, 14. Ribbon model shows the binding pocket structure of Human peroxiredoxin 5 with 4. Hydrogen bond between compounds and amino acids are shown as green dash lines, hydrophobic interaction are shown as pink lines.

**Table 5:** Molecular docking value of isolated compounds (1–5) against *human* peroxidoxin (PDB ID: 1HD2)

Compounds	Affinity (kcal/mol)	H-bond	Residual Amino acid Interactions	
			Hydrophobic/Pi-Cation/Pi-Anion/ Pi-Alkyl interactions	Van-der Walls interactions
**1**	− 5.2	Thr-147	Leu-149	Thr-44, Pro-45, Gly-46, Thr-50, Ile-119, Phe-120
**2**	− 5.1	Gly-46	–	Cys-47, Thr-44, Pro-40, Leu-116, Phe-120, Arg-127, Gly-148, Leu-149
**3**	− 4.5	Asn-76, Arg-124	Pro-40, Pro-45, Leu-116, Ile-119, Phe-120	Cys-47, Thr-44, Gly-41, Ala-42, Arg-127, Thr-147
**4**	− 4.8	Thr-147	Pro-45	Thr-44, Ala-42, Leu-116, Ile-119, Phe-120, Arg-124
**5**	− 4.9	–	Cys-47, Pro-45, Ile-119, Phe-120	Pro-40, Phe-43, Ala-42, Asn-76, Leu-116, Arg-124, Thr-147
**Ascorbic Acid**	− 4.9	Cys-47, Thr-44, Gly-46, Thr-147	Pro-40, Pro-45, Phe-120, Arg-127, Leu-149	–

### *In-Silico* pharmacokinetics (Drug-likeness) analysis

The SwissADME prediction outcome showed that the isolated compounds (**2–5**) satisfy Lipinski's rule of five with zero violations (Table 6) [31]. As per the Lipinski’s rule of five, the molecular weight of the molecules should be less than 500 and the lipophilicity (iLogP) values should be less than 5. The compound **1** violates these two rules. The Kp values of all molecules are within the range of (− 2.24 to − 3.96 cm/s) with ciprofloxacin (− 8.98 cm/s) inferring low skin permeability (Table 7). The predicted logP values revealed that compounds **2**, **3** and **5** have optimal lipophilicity (ranging from 2.4 to 4.7). The SwissADME prediction parameters showed that compounds **2** and **3** have high gastrointestinal (GI) absorption, blood brain barrier (BBB) permeation and compounds **1** and **2** are substrate of permeability glycoprotein (P-gp). These prediction results indicate that isolated compound **2** can be better active pharmacological agent compared to other isolated compounds.

**Table 6:** In silico drug-likeness predictions of isolated compounds computed by SwissADME

Compounds	Formula	Mol. Wt. (g/mol)	NHD	NHA	NRB	TPSA (A^°2^)	LogP iLogP)	Lipinski’s rule of Five Violation
**1**	C_46_H_66_O_6_	715.01	1	6	10	105.58	5.19	Yes
**2**	C_22_H_30_O_4_	358.47	1	4	0	71.44	2.4	0
**3**	C_14_H_28_O_2_	228.37	1	2	12	37.3	3.32	0
**4**	C_29_H_48_O	412.69	1	1	6	20.23	5.1	Yes
**5**	C_29_H_48_O	412.69	1	1	5	20.23	4.79	0
	Ciprofloxacin	401.45	2	9	5	136.13	0.963	0

NHD: Number of Hydrogen donor; NHA: Number of Hydrogen acceptor; NRB: Number of rotatable bonds; TPSA: total polar surface area

**Table 7:** ADME predictions of compounds **1–5**, computed by SwissADME and PreADMET

Cpds	Formula	Skin Permeation Value (log Kp) cm/s	GI Absorption	BBB Permeability	Inhibitor Interaction (SwissADME/PreADMET)					
					P-gp substrate	CYP1A2 inhibitor	CYP2C19 inhibitor	CYP2C9 inhibitor	CYP2D6 inhibitor	CYP3A4 inhibitor
**1**	C_46_H_66_O_6_	− 3.96	Low	No	Yes	No	No	No	No	No
**2**	C_22_H_30_O_4_	− 7.25	High	Yes	Yes	No	No	No	No	No
**3**	C_14_H_28_O_2_	− 3.35	High	Yes	No	Yes	No	No	No	No
**4**	C_29_H_48_O	− 2.24	Low	No	No	No	No	Yes	No	No
**5**	C_29_H_48_O	− 2.92	Low	No	No	No	No	No	No	No
	Ciprofloxacin	− 8.98	High	No	Yes	Yes	No	No	No	No

GI: Gastro-Intestinal; BBB: Blood Brain Barrier; P-gp: P-glycoprotein; CYP: Cytochrome-P

## Experimental

### General experimental procedure

Melting point was determined in capillary tube with a digital electrothermal melting point apparatus. Analytical TLC was run on a 0.25 mm thick layer of silica gel GF254 (Merck) on aluminum plate. Spots were detected either by observation under UV light (254 nm) or spraying with vanillin/H_2_SO_4_. Column chromatography was performed using silica gel (230–400 mesh) Merck. Samples were applied on column by adsorbing on silica gel. Solvent was removed using rotavapor. The UV–Vis spectral measurements were done using UV–Vis on T 60 U spectrophotometer (PG instruments, UK) equipped with deuterium and tungsten lamps. The IR spectra of compounds were recorded using a Perkin-Elmer BX Spectrometer (400–4000 cm^−1^) as KBr pellets. NMR spectra were recorded using Bruker Avance 400 spectrometer operating at 400 MHz.

Plant material: The roots of *C. prophetarum* were collected in April, 2019 from Dheka Dima, Borena zone, Oromia regional state, Ethiopia following the guideline proposed by Wondafrash (2008) [32]. The plant was collected after getting written consent from the local authority. It was authenticated by Mr. Shambel Alemu and voucher specimen WA-001 for *C. prophetarum* was deposited at the National Herbarium of Addis Ababa University, Ethiopia.

### Extraction and isolation of compounds from the roots of *C. prophetarum*

The air dried powdered root of *C. prophetarum* (400 g) was successively extracted by maceration with each 2 L of *n*-hexane, CHCl_3_ and MeOH at room temperature for 72 hrs, filtrated and concentrated at reduced pressure using rotary evaporator at 35 °C to afford their corresponding extracts. The *n*-hexane (2 g) and EtOAc extract (2 g) shown similar TLC profile were mixed together, adsorbed and fractionated over silica gel (150 g) column chromatography with *n*-hexane: EtOAc of increasing polarity to give 25 fractions, each 300 mL. The first three fractions were eluted with *n*-hexane. The next nineteen fractions (Fr4-Fr23) were eluted with 5% increament of EtOAc in *n*-hexane. Finally elution was completed with EtOAc. Fr5 eluted with *n*-hexane: EtOAc (9:1), Fr6 eluted with *n*-hexane: EtOAc (85:15) and Fr9 eluted with *n*-hexane: EtOAc (7:3) were identified as compound 3, 4 and 5, respectively. Compound 1 was identified after purification of Fr13 using silica gel column chromatography. Fr15, eluted with *n*-hexane: EtOAc (2:3), was identified as compound 2.

### Antibacterial activity

Antibacterial activities of the crude extracts and isolated compounds were investigated in vitro using Agar well diffusion method in Mueller–Hinton brothagainst four bacterial strains such as *S. aureus, B. subtilis, E. coli* and *S. typhi* following reported procedure [33]. Appropriate colonies of required bacterial strains were selected and picked using an inoculating loop and inoculum suspension was prepared and standardized with 0.5 McFarland standards to afford 5 × 10^5^ CFU/mL. The extracts and isolated compounds were dissolved in DMSO to obtain 2.5, 5 and 7.5 mg/mL. The surface of the medium plate was inoculated with standardized suspension and the discs containing crude extract and isolated compounds of different concentrations (2.5, 5 and 7.5 in mg/mL), chloramphenicol as standard drugs (2.5 mg/mL) and DMSO as negative control were applied within 15 min of inoculation. Each was then incubated in ambient air at 35 °C for 16–18 h. The samples were analyzed in triplicates and standard deviation was calculated. Zones of inhibitions were measured from the back of the plate by ruler and interpreted as inactive active and very active following literature guide. Antibacterial activity (*x*) was then characterized and classified based on the inhibition growth zone diameters and described as slight (*x* < 4 mm diameter), medium (*x* = 4–8 mm), high (*x* = 8–12 mm), and very high (*x* > 12 mm) [34].

### Molecular docking studies of the isolated compounds

AutoDock Vina with standard protocol was used to dock the proteins (PDB ID: 6F86 and PDB ID: 1HD2) and isolated chemical constituents (**1–5**) into the active site of proteins [35, 36]. The chemical structures of compounds **1**–**5** were drawn using ChemOffice tool (Chem Draw 16.0) assigned with proper 2D orientation, and energy of each molecule was minimized using ChemBio3D. The energy minimized ligand molecules were then used as input for AutoDock Vina, in order to carry out the docking simulation. The crystal structure of receptor molecule *E. coli* gyrase B (PDB ID: 6F86) and Human peroxiredoxin 5 (PDB ID: 1HD2) were downloaded from protein data bank. The protein preparation was done using the reported standard protocol [37] by removing the co-crystallized ligand, selected water molecules and cofactors, the target protein file was prepared by leaving the associated residue with protein by using Auto Preparation of target protein file Auto Dock 4.2 (MGL tools1.5.7). The graphical user interface program was used to set the grid box for docking simulations. The grid was set so that it surrounds the region of interest in the macromolecule. The docking algorithm provided with Auto Dock Vina was used to search for the best docked conformation between ligand and protein. During the docking process, a maximum of nine conformers were considered for each ligand. The conformations with the most favorable (least) free binding energy were selected for analyzing the interactions between the target receptor and ligands by Discovery studio visualizer and PyMOL. The ligands are represented in different color, H-bonds and the interacting residues are represented in stick model representation.

### *In-silico* drug-likeness predictions

*In-Silico* Drug-likeness is a prediction that concludes whether a particular pharmacological agent has properties consistent with being an orally active drug. This prediction is based on an already established concept by Lipinski et al, called Lipinski rule of five [31]. The structures of isolated compounds (**1–5**) were changed to their canonical simplified molecular input line entry system (SMILE) then submitted to SwissADME tool to estimate in silico pharmacokinetic parameters and other molecular properties based on the methodology reported by Amina et al. [38]. The analyses of the compounds were compared with that of clinical drug (ciprofloxacin), and only compounds without violation of any of the screenings were used for the molecular docking analysis.

### Antioxidant activities of the extracts and isolated compounds

The antioxidant activities of the extracts and constituents of the roots of *C. prophetarum* were studied using DPPH and ferric thiocyanate methods.

### DPPH Radical Scavenging Assay

The DPPH radical scavenging activities of the extracts and isolated compounds were evaluated using DPPH assay following procedure indicated in Rivero-Perez et al. [39] with slight modification. The extracts and isolated compounds from the roots of *C. prophetarum* were dissolved in methanol to afford 1 mg/mL. Each was serially diluted in methanol to give concentration of 500, 250, 125 and 62.5 µg/mL. To 1 mL of each concentration, 4 mL DPPH (0.1 mM DPPH in MeOH) was added to make 100, 50, 25 and 12.5 µg/mL solutions. Then all the samples prepared were incubated in an oven at 37 °C for 30 min. and then absorbance was recorded at 517 nm using UV–Vis spectrophotometer. The percentage inhibition was calculated using the formula [40].

$$ \% {\text{ Inhibition}}\, = \,\left( {{\text{A}}_{{{\text{control}}}} {-}{\text{A}}_{{{\text{extract}}}} } \right)/{\text{A}}_{{{\text{control}}}} \, \times \,{1}00 $$

where A_control_ is the absorbance of DPPH solution and A_extract_ is the absorbance of the test sample (DPPH solution plus sample).

### Ferric thiocyanate method

The extracts and constituents of the roots of *C. prophetarum* were also evaluated for their antioxidant activity using ferric thiocyanate methods [41]. In this regards, each 0.1 mg the extracts and pure compounds of the roots of *C. prophetarum*, 100 µL of linoleic acid, EtOH (5 mL) and phosphate buffer (5 mL, 0.05 M, pH = 7) in water were separately added in to a vial and incubated at 40 °C in an oven. After 24 h, 0.1 mL from each were taken and added in to a vial containing 75% aqueous EtOH (7 mL), 30% of NH_4_SCN (0.15 mL) and 0.15 mL of 0.02 M FeCl_2_ in 3.5% HCl. Each was then subjected to UV–Vis spectrophotometery to record the absorbance at 500 nm. Absorbance of the blank and ascorbic acid were done in the same fashion. The percentage inhibition using ferric thiocyante method is calculated according to the following formula.

$$ {\text{Percentage inhibition}}\, = \,{1}00 - \left( {\frac{{{\text{As}}}}{{{\text{Ab}}}}\; \times \;100} \right)\% $$

where as is assorbance of the sample and Ab is absorbance of the blank [28].

### Statistics data analysis

The antimicrobial analysis data generated by triplicate measurements were reported as mean ± standard deviation. GraphPad Prism version 5.00 for Windows was used to perform the Analysis (GraphPad Software, San Diego California USA, www.graphpad.com). Groups were analyzed for significant differences using a linear model of variance analysis (ANOVA) test for comparisons was performed, with significance accepted for p < 0.05 (Additional file 1).

## Conclusion

The present study identified five compounds from the root extracts of *C. prophetarum*, of which two are novel cucurbitacins (**1, 2**). The in vitro antibacterial activity of the hexane and methanol extracts was better than the activity displayed by the isolated compounds. This is probably due to the synergistic effects of the constituents present in the root extract. The in silico molecular docking study results showed that, compounds **2** and **3** have minimum binding energy and have good affinity toward the active pocket, thus, they may be considered as good inhibitor of DNA gyrase B. Furthermore, the “drug-likeness” and ADMET prediction of compounds **2–5** nearly showed compliance with the Lipinski rule, with good absorption, distribution, metabolism, and excretion generally. The radical scavenging and anti-lipid peroxidation activities of the extracts were better than the isolated compounds. This may be attributed to the presence of phenolics and flavonoids as minor constituents in the extracts of these species. The binding affinity the isolated compounds with human peroxidoxin (PDB ID: 1HD2) are matching with antioxidant analysis. Therefore, the in vitro antibacterial activity and molecular docking analysis suggest the potential use of the isolated compounds as medicine which corroborates the traditional use of the roots of *C. prophetarum*.

## Supplementary Information

**Additional file 1.** The 1D and 2D NMR spectra for the new compounds 1 and 2 are included within supplementary materials (Additional file 1).

## Data Availability

The datasets supporting the findings of this article are presented in the main manuscript. Plant materials used in the study have been identified by Mr. Shambel Alemu and voucher specimen was deposited at the National Herbarium of Addis Ababa University, Ethiopia. AutoDock Vina conformations for compounds against LasR and DNA gyraseB binding domain and the NMR spectra of the known compounds 3–5 can be accessed from the corresponding author on reasonable request.
