# Supplementary material for: Antibacterial and antioxidant activities of extracts and isolated compounds from the roots extract of Cucumis prophetarum and in silico study on DNA gyrase and human peroxiredoxin 5
Source: BMC Chem. 2021 May 6;15(1):32. doi: 10.1186/s13065-021-00758-x (PMC8103605; doi:10.1186/s13065-021-00758-x)

Antibacterial and Antioxidant Activities of Extracts and Isolated Compounds from the Roots Extract of *Cucumis prophetarum* and *In Silico* Study on DNA Gyrase and Human Peroxiredoxin 5

Wario Galma, Milkyas Endale, Emebet Getaneh, Rajalakshaanan Eswaramoorthy, Temesgen Assefa, Yadessa Melaku*

Corresponding author: Dr. Yadessa Melaku

The NMR spectra used to establish the structures of the two novel compounds isolated in this work are depicted as supporting information 1-12

Supporting information 1: ^{1}H NMR spectrum of compound 1

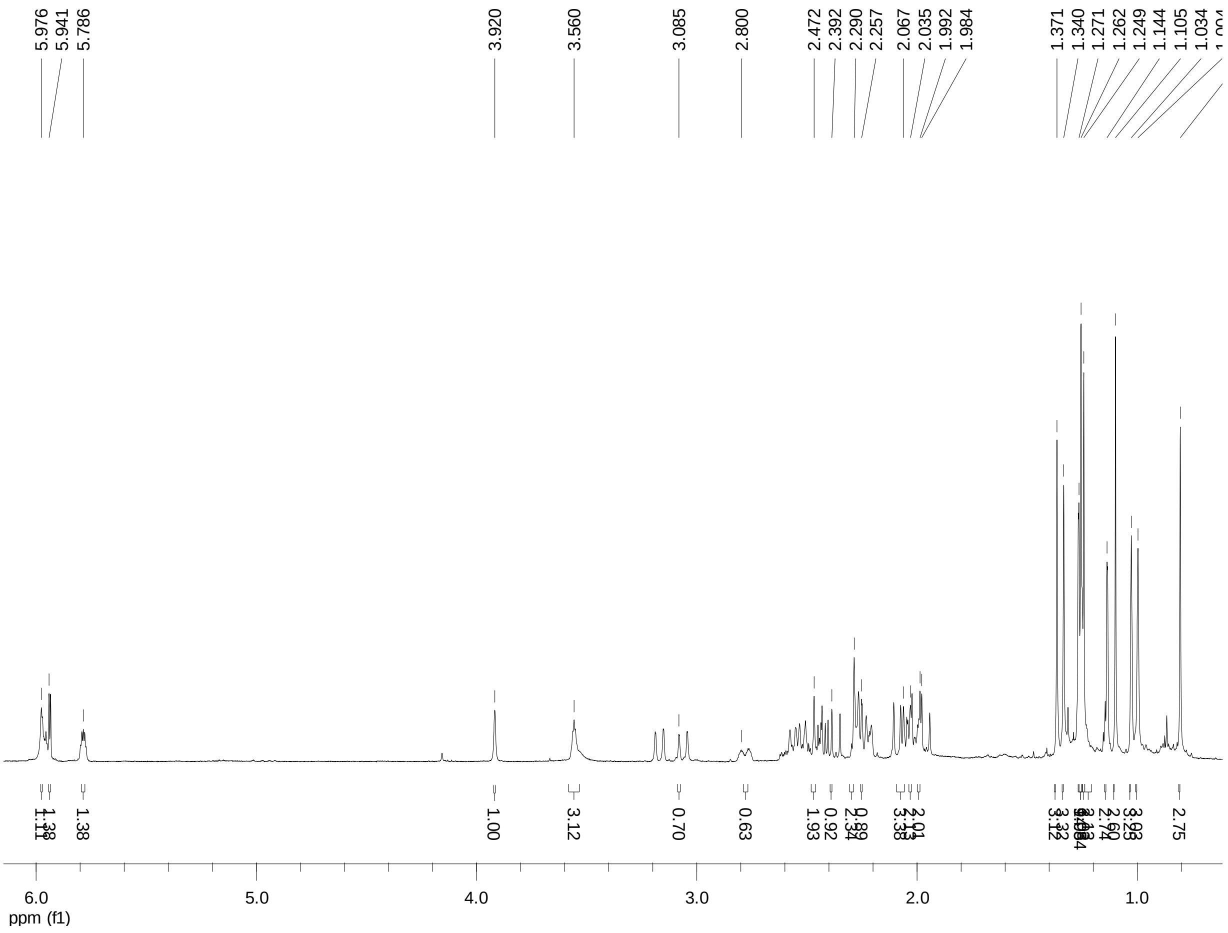

Supporting information 2: ^{13}C NMR spectrum of compound 1

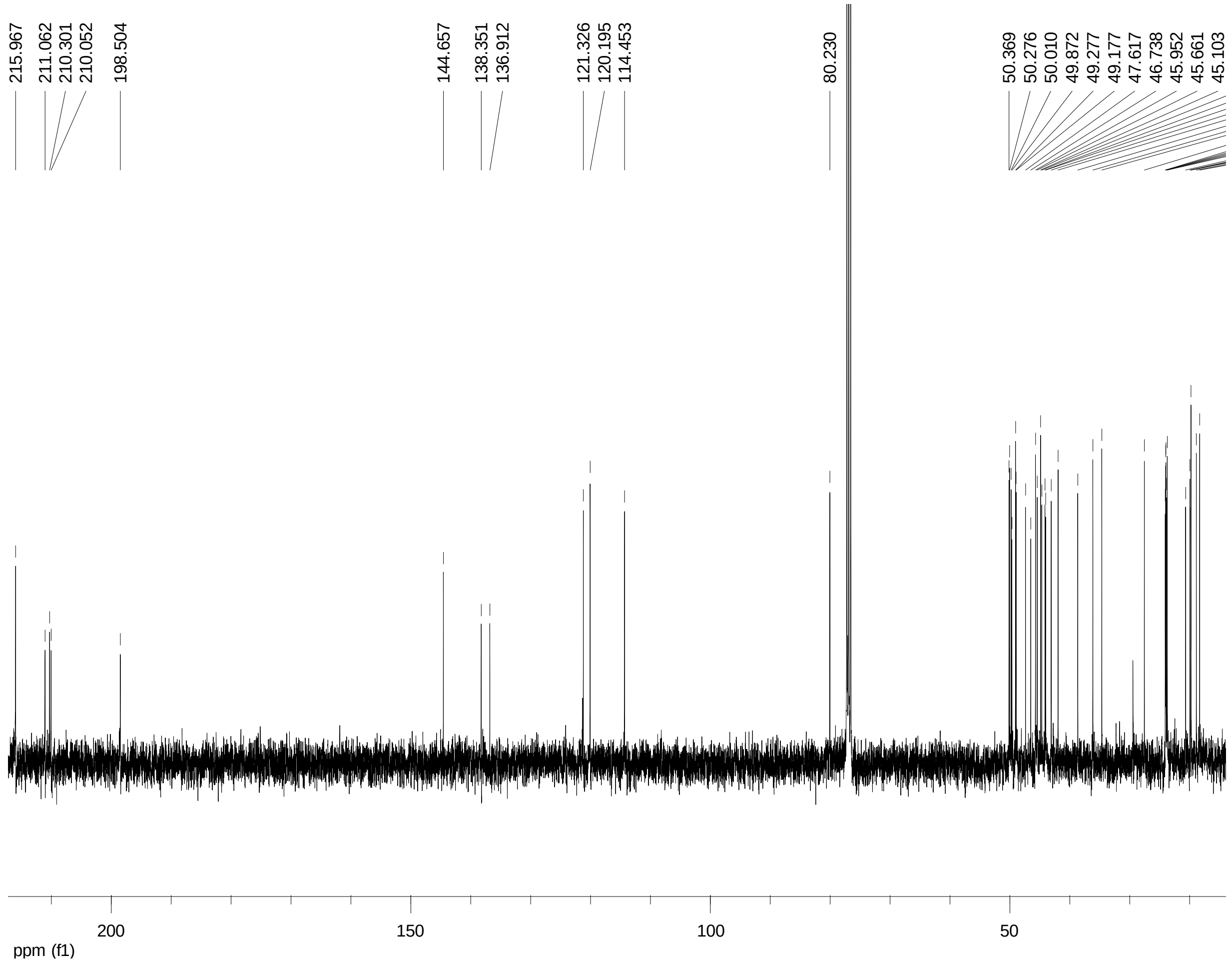

Supporting information 3: DEPT-135 NMR spectrum of compound 1

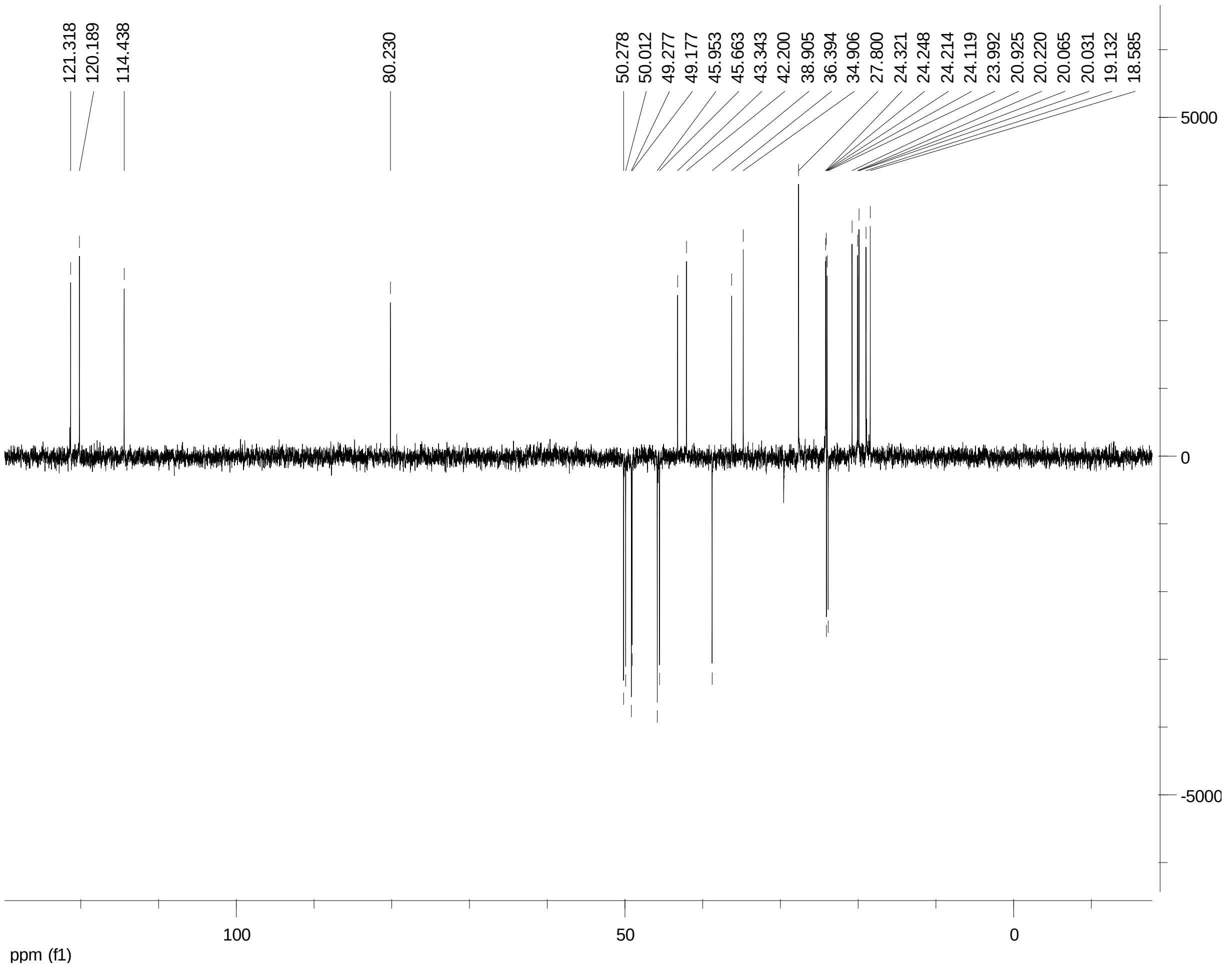

Supporting information 4: COSY spectrum of compound 1

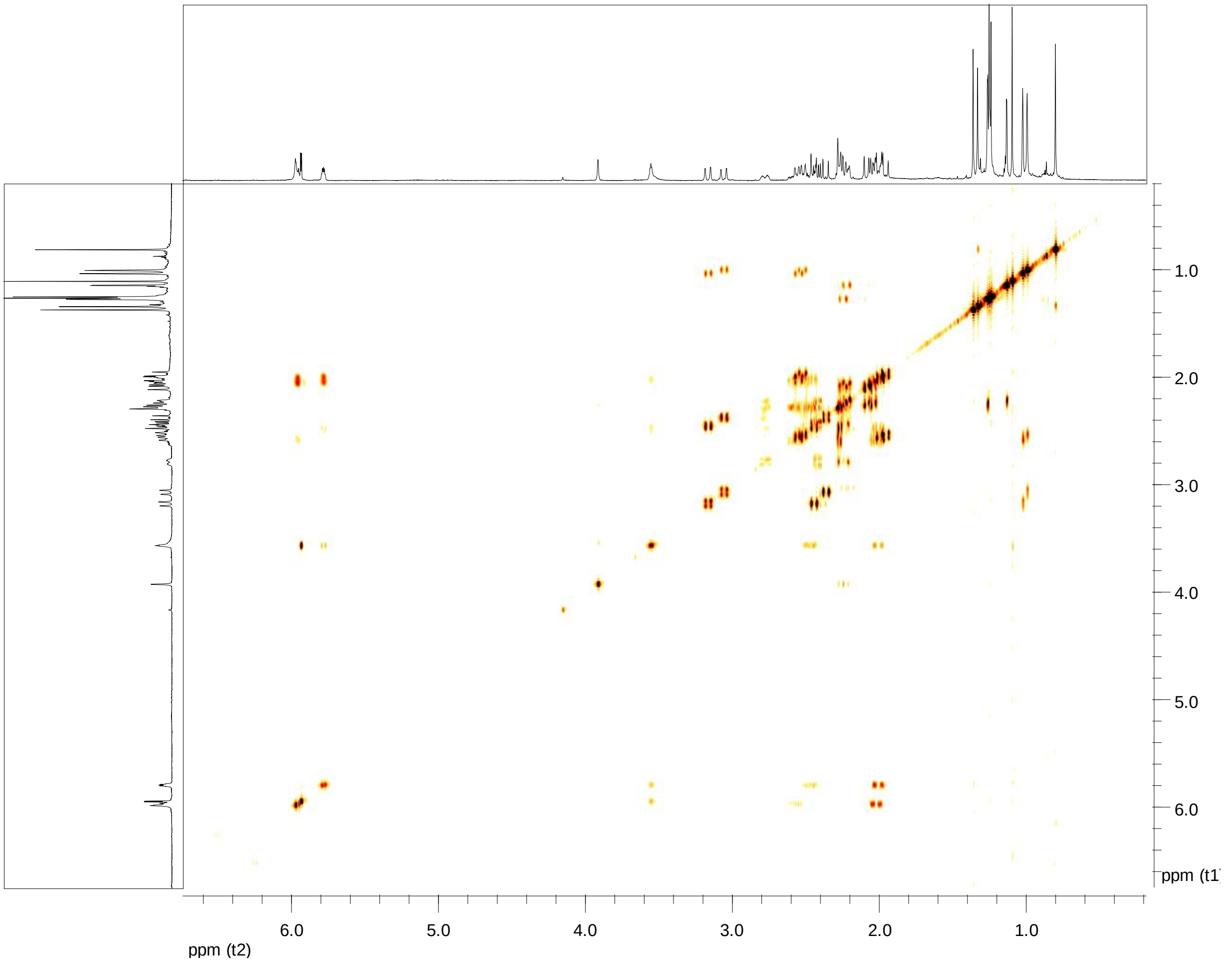

Supporting information 5: HSQC spectrum of compound 1

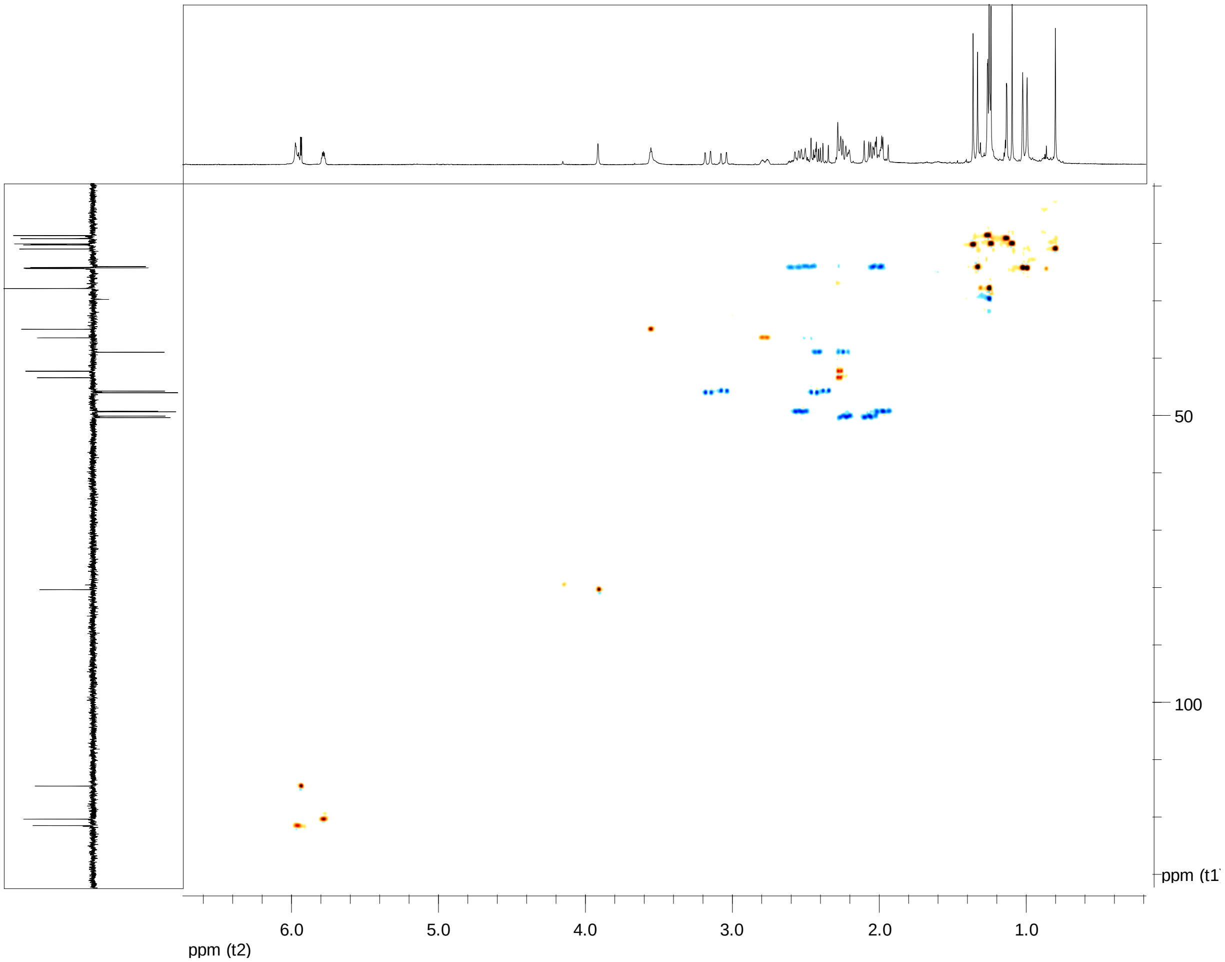

Supporting information 6: HMBC spectrum of compound 1

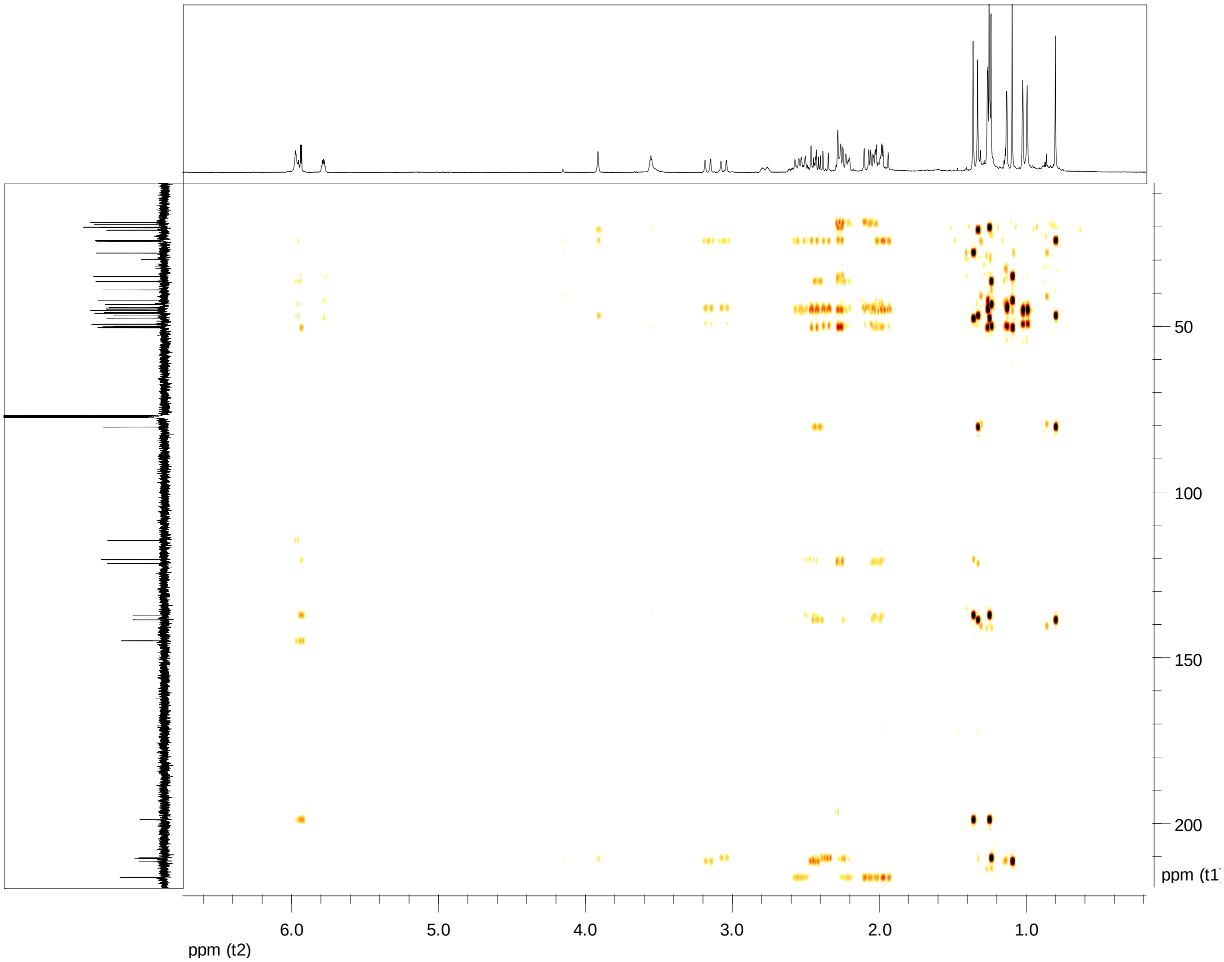

Supporting information 7: ^{1}H-NMR spectrum of compound 2

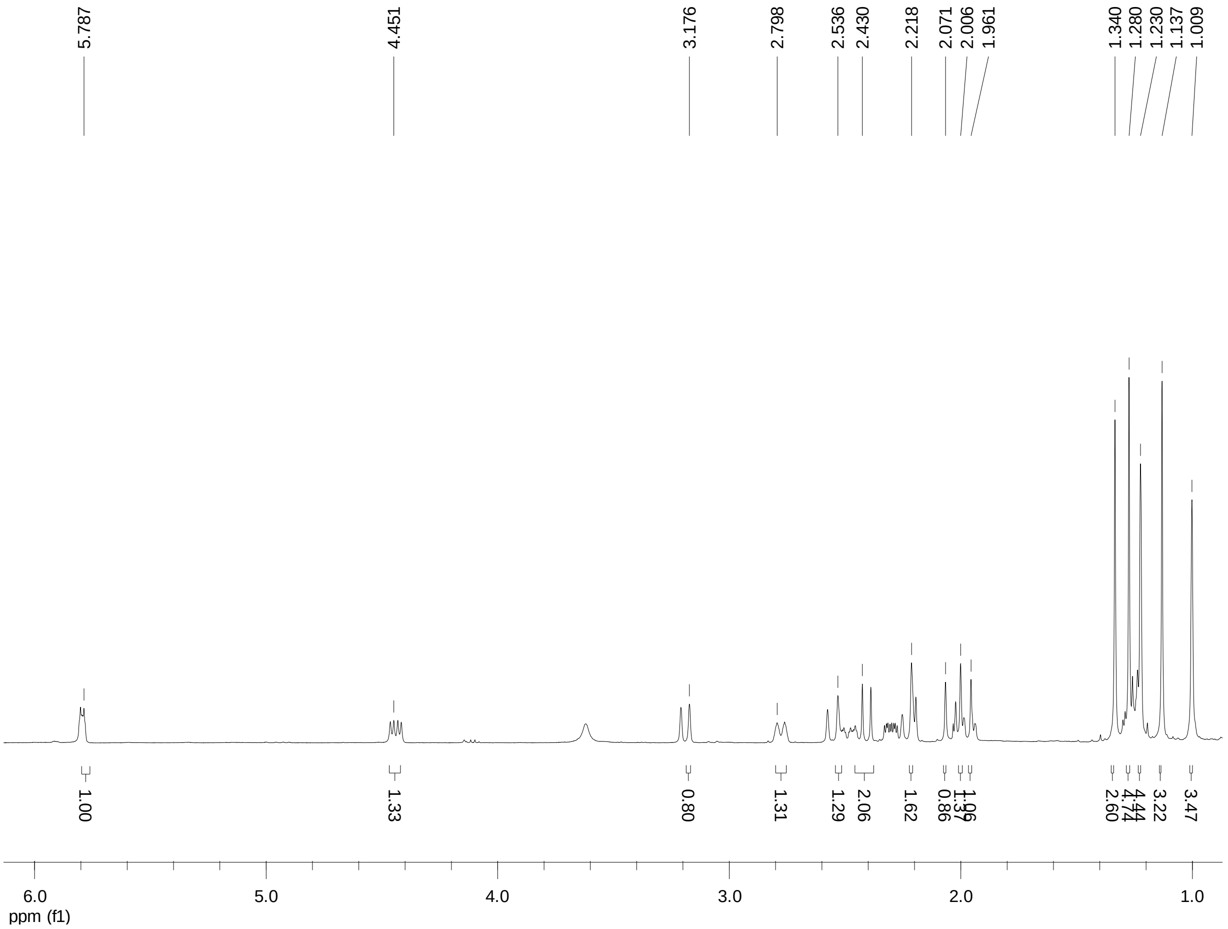

Supporting information 8: ^{13}C-NMR spectrum of compound 2

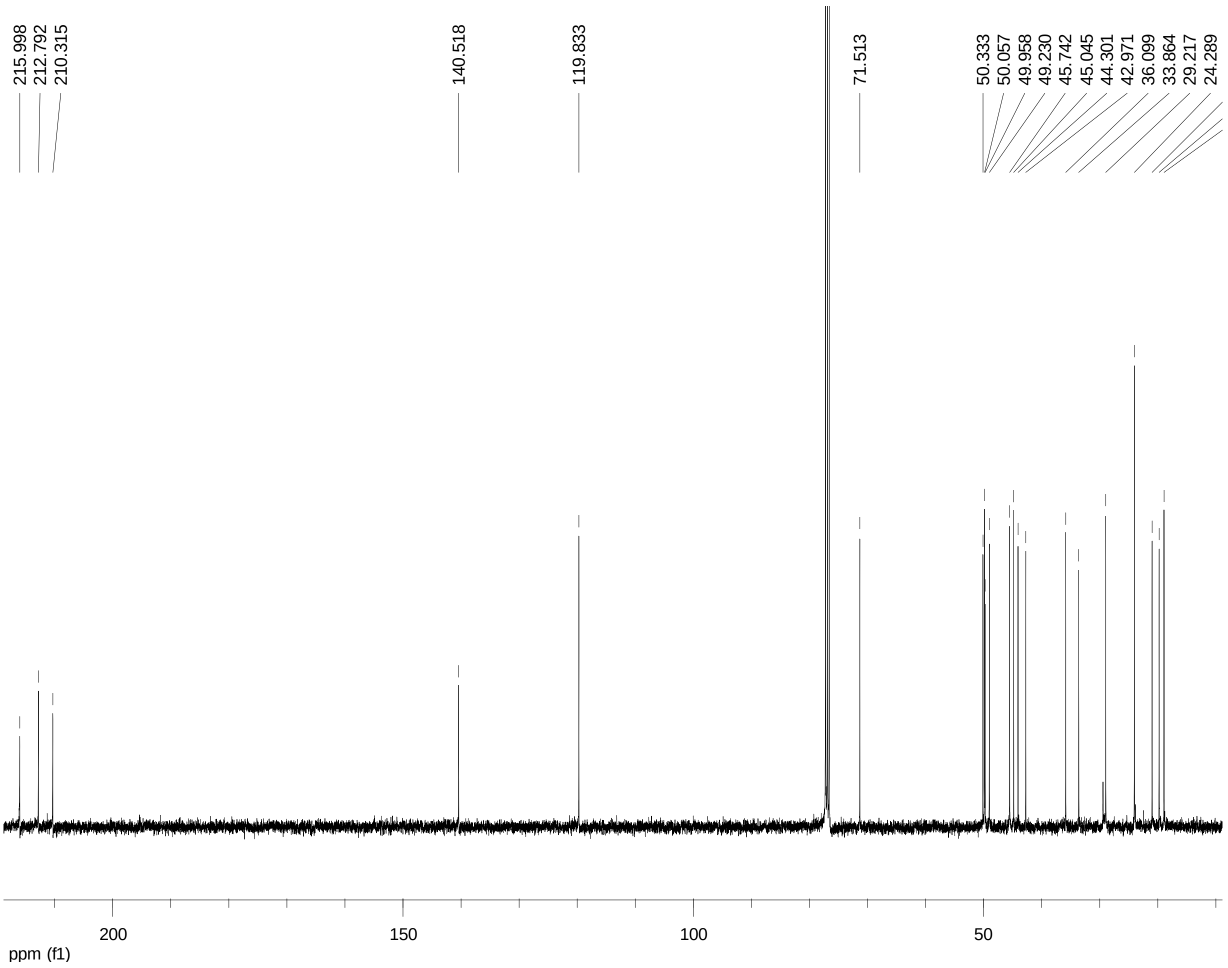

Supporting information 9: DEPT-135 spectrum of compound 2

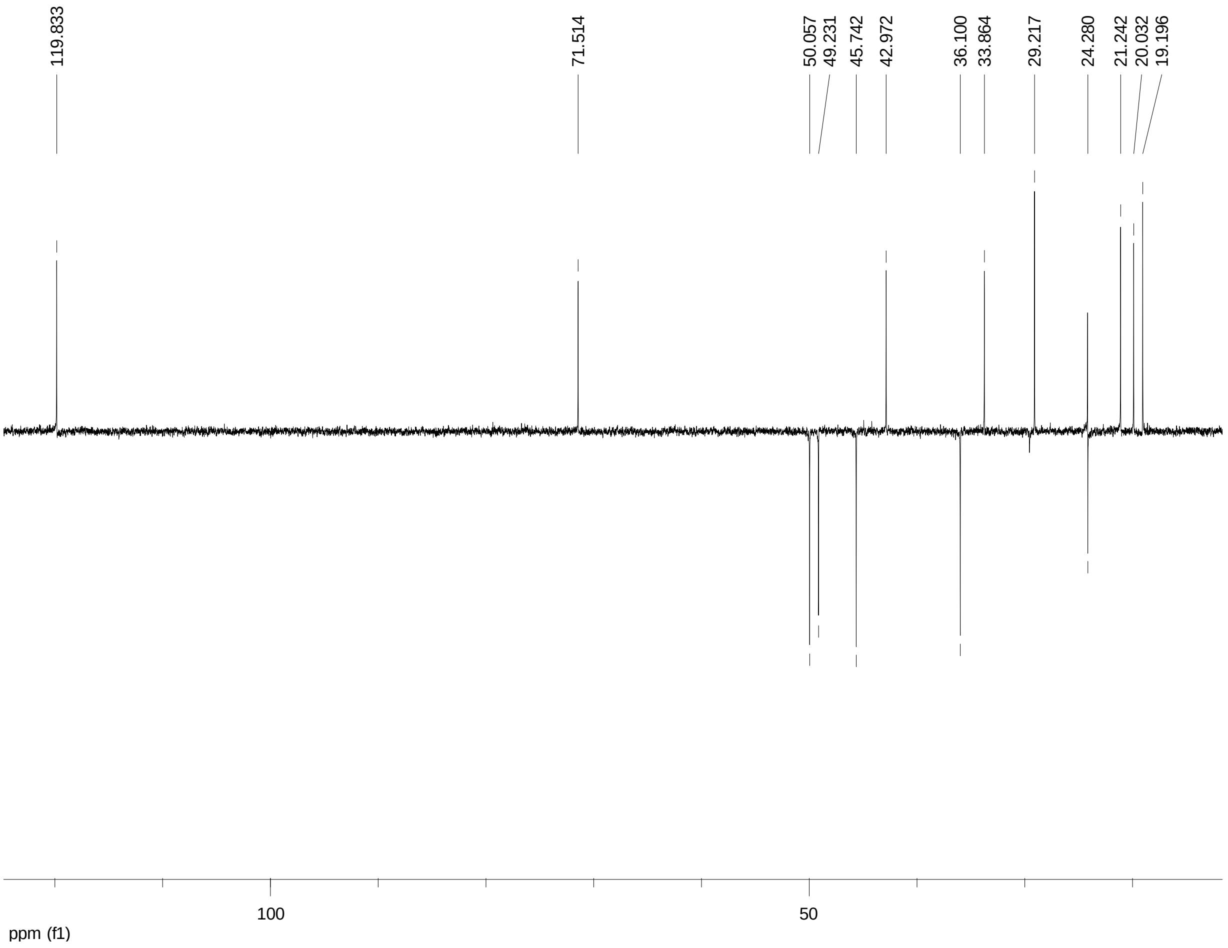

Supporting information 10: COSY spectrum of compound 2

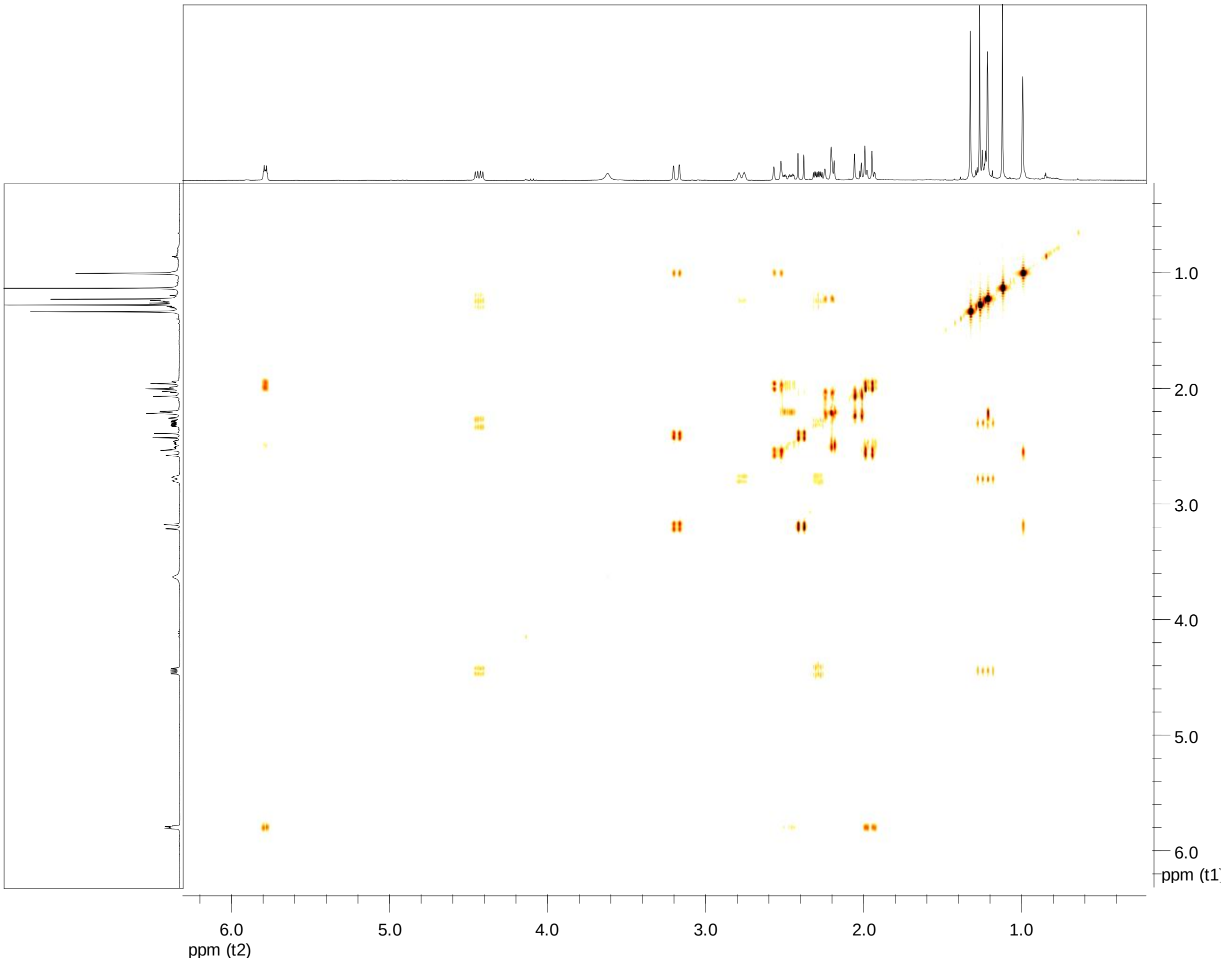

Supporting information 11: HSQC spectrum of compound 2

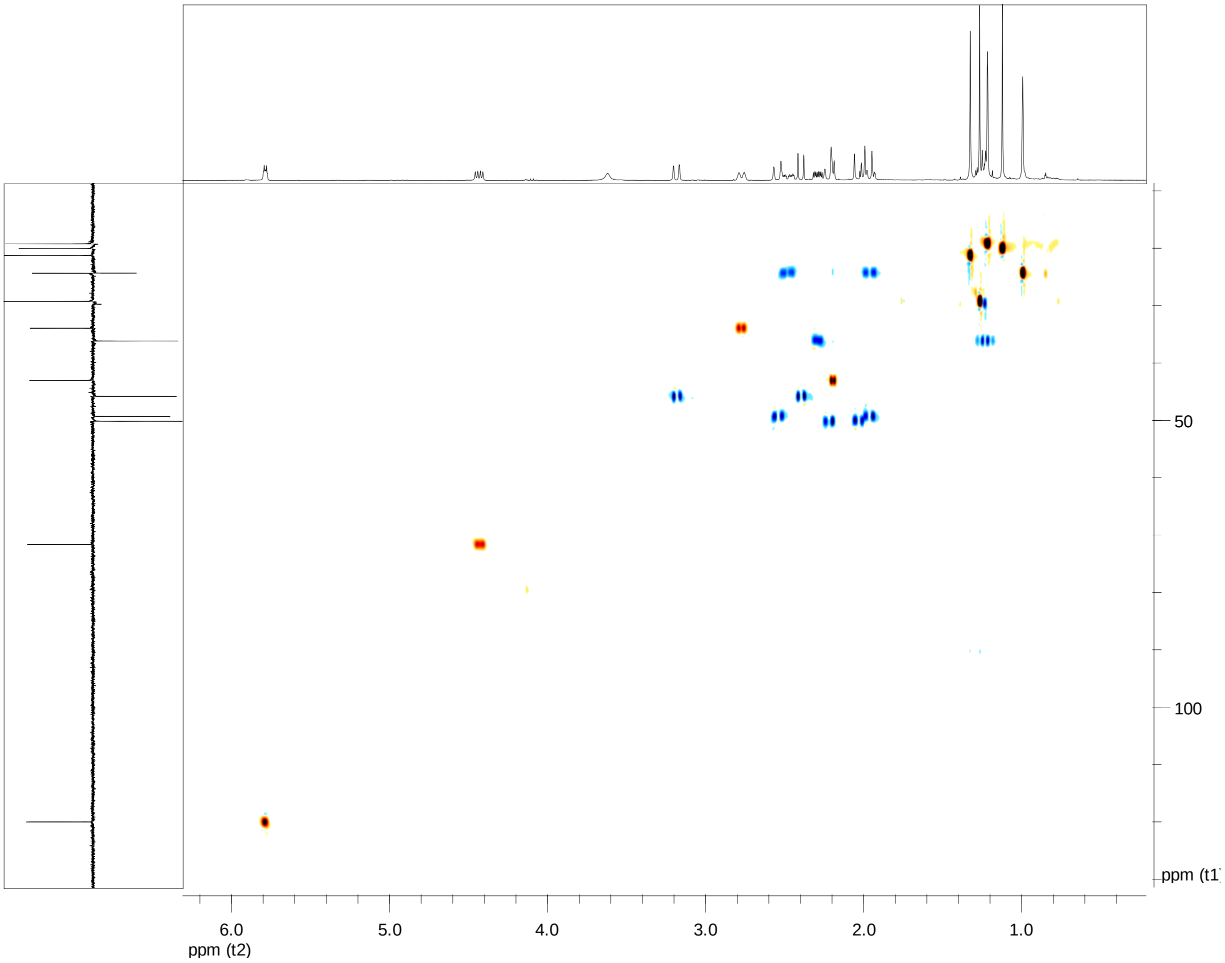

Supporting information 12: HMBC spectrum of compound 2

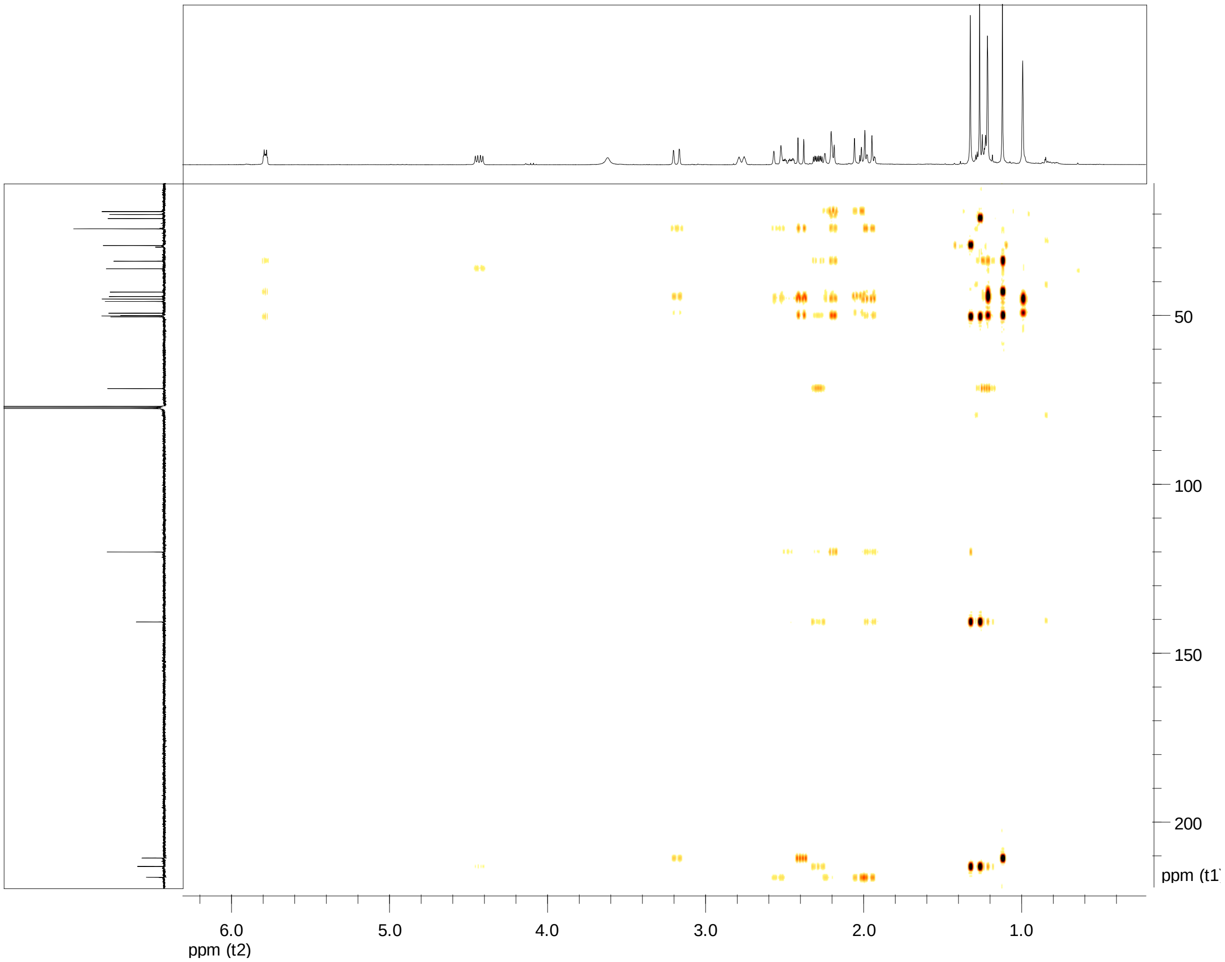

Supplement: Supplementary file 1 — Additional file 1. The 1D and 2D NMR spectra for the new compounds 1 and 2 are included within supplementary materials (Additional file 1). [file 13065_2021_758_MOESM1_ESM.pdf]
